# A New Single Gene Differential Biomarker for *Mycobacterium tuberculosis* Complex and Non-tuberculosis Mycobacteria

**DOI:** 10.3389/fmicb.2019.01887

**Published:** 2019-08-13

**Authors:** Lei Zhou, Cuidie Ma, Tongyang Xiao, Machao Li, Haican Liu, Xiuqin Zhao, Kanglin Wan, Ruibai Wang

**Affiliations:** ^1^State Key Laboratory for Infectious Disease Prevention and Control, National Institute for Communicable Disease Control and Prevention, Chinese Center for Disease Control and Prevention, Beijing, China; ^2^College of Pharmacy, Guizhou University, Guiyang, China; ^3^College of Life Science and Technology, Beijing University of Chemical Technology, Beijing, China

**Keywords:** *Mycobacterium*, non-homologous DNA end-joining, polymorphism, biomarker, non-tuberculosis mycobacteria, non-sequencing

## Abstract

**Background:**

Tuberculosis (TB) and non-tuberculous mycobacteriosis are serious threats to health worldwide. A simple non-sequencing method is needed for rapid diagnosis, especially in less experienced hospitals, but there is no specific biomarker commonly used for all mycobacteria. The *ku* gene of the prokaryotic error-prone non-homologous end joining system (NHEJ) has the potential to be a highly specific detection biomarker for mycobacteria.

**Methods:**

A total of 7294 mycobacterial genomes and 14 complete genomes of other families belonging to *Corynebacteriales* with *Mycobacteriaceae* were downloaded and analyzed for the existence and variation of the *ku* gene. *Mycobacterium tuberculosis* complex (MTBC) and non-tuberculosis mycobacteria (NTM)- specific primers were designed and the actual amplification and identification efficiencies were tested with 150 strains of 40 *Mycobacterium* species and 10 kinds of common respiratory pathogenic bacteria.

**Results:**

The *ku* gene of the NHEJ system was ubiquitous in all genome sequenced *Mycobacterium* species and absent in other families of *Corynebacteriales*. On the one hand, as a single gene non-sequencing biomarker, its specific primers could effectively distinguish mycobacteria from other bacteria, MTBC from NTM, which would make the clinical detection of mycobacteria easy and have great clinical practical value. On the other hand, the sequence of *ku* gene can effectively distinguish NTM to species level with high resolution.

**Conclusion:**

The Ku protein existed before the differentiation of *Mycobacterium* species, which was an important protein involved in maintaining of the genome’s integrity and related to the special growth stage of mycobacteria. It was rare in prokaryotes. These features made it a highly special differential biomarker for *Mycobacterium*.

## Introduction

*Mycobacterium* is a genus of over 190 species and 13 subspecies. Apart from the causative agents, the *Mycobacterium tuberculosis* complex (MTBC) and *Mycobacterium leprae*, the other members of this genus are grouped together and termed non-tuberculosis mycobacteria (NTM). Both tuberculosis (TB) and non-tuberculous mycobacteriosis pose serious threats to health worldwide, especially with the increase of multi-drug and pan-drug-resistant strains.

Mycobacteria have completely different culture characteristics and therapeutic antibiotics from other bacteria. A preliminary discrimination of the infection as mycobacteriosis, TB or NTM, or a mixed infection of these two kinds of mycobacteria, will make the subsequent culture more directed and help improve the isolation/culture rate and appropriately administer clinical medication to reduce transmission more effectively, especially for less-experienced hospitals. This method should be simple, fast, accurate and culture-free.

Compared with the relatively easy diagnosis of tuberculosis, the diagnosis of non-tuberculosis is more dependent on culture and biochemical tests or by the exclusion that negative TB detection in smear/culture of acid-positive samples. Because of the cumbersome procedure, isolation, cultivation and identification of NTM is not actually done in many hospitals in China. The incidence and disease burden of NTM are continuously increasing in many regions, and the prevalence of NTM in aged people, human immunodeficiency virus (HIV)-infected patients and those with severely damaged immune systems is significant, even more than that of TB (Wang et al., 2014; Halstrom et al., 2015; Mortaz et al., 2018). Therefore, the rapid diagnosis of NTM is also a prominent problem. It is necessary to find a better single-gene biomarker, which can be used for both of MTBC and NTM identification.

Mycobacteria have three DNA double-strand break repair pathways, which include the NHEJ system required for the CRISPR/Cas9 system in the second step (Pitcher et al., 2006). The NHEJ system is absent in most prokaryotic cells. To date, eukaryotic NHEJ homologs have only been identified in *M. smegmatis*, *M. tuberculosis*, and *Bacillus subtilis* (*Bs*). Furthermore, prokaryotic NHEJ is a much simpler system that needs only two key proteins, Ku and ligase D (LigD) (Gong et al., 2005; Korycka-Machala et al., 2006; Shuman and Glickman, 2007; Gupta et al., 2011; Zheng et al., 2017). The Ku protein exists as a homodimer and preferentially binds to dsDNA ends (Weller et al., 2002). LigD is an adenosine triphosphate (ATP)-dependent DNA ligase that contains polymerase and nuclease domains, which facilitates the joining of long linear DNA molecules with different incompatible ends (Della et al., 2004). The rarity of the NHEJ system in bacteria hints that it may be developed into a *Mycobacterium* specific detection biomarker. Although, the NHEJ system has been confirmed existing in the *M. tuberculosis* strain H37Rv (Rv0937c and Rv0938 encoded) (Doherty et al., 2001; Weller et al., 2002), its distribution, especially the distribution of the Ku protein that could specifically stimulate LigD and suppress homologous recombination (Della et al., 2004) in other *Mycobacterium* species has not yet been elucidated. In this study, we analyzed *Mycobacterium* genome data submitted in GenBank before September 2018 to explore the existence of the *ku* gene in the *Mycobacterium* genus and determine its applicability for *Mycobacterium* identification.

## Materials and Methods

### Genomic Data

We downloaded a total of 7294 genomes from 139 definite species, seven subspecies and five variants of *Mycobacterium* from the NCBI’s FTP site submitted before September 2018, including 5245 genomes of *M. tuberculosis*, 1376 genomes of *M. abscessus*, 152 genomes of *M. avium*, and 70 *M. tuberculosis* variant *bovis* (Supplementary Table 1).

### Sequence Extraction and Analysis

All the regions annotated as *ku* or *mku* and/or homologous to Rv0937c of *M. tuberculosis* were extracted from the genomes. Sequence alignments and comparisons were performed using the MEGA program version 6.0 (Tamura et al., 2013). Sequences were aligned on ClustalW using a gap opening penalty of 15 and a gap extension penalty of 6.66. Maximum likelihood trees were drawn. In each *Mycobacterium* species/variant, every *ku* sequence with even a nucleotide difference was defined as a genotype and listed in Supplementary Table 1 with a representative sequence. The IS*6110* and *rpoB* genes were analyzed much as the *ku* gene was, and Supplementary Table 2 lists the genotypes of the *rpoB* gene.

### Primer Designed and PCR Amplification

Primers were designed using Oligo 6.0 and followed the general design principle of PCR primer. Simulated PCRs were performed using the Analyze Mix Wizard of Clone Manager Professional 9.0. The 379 *ku* genotype sequences were added as molecules in the mix.

In actual PCR amplification, the boiled DNA temples of *M. tuberculosis* H37Rv, *M. bovis* 93006 and 43 American Type Culture Collection (ATCC) NTM strains belonging to 40 species were used (Table 1). In addition, clinical isolates including 42 *M. tuberculosis* strains, 10 *M. abscessus* strains, 10 *M. marseillense* strains, 10 *M. avium* strains, 10 *M. kansasii* strains and 23 strains of 10 kinds of common respiratory pathogenic bacteria (Table 1) were also tested. PCR amplification condition was 5 min at 95°C followed by 30 cycles of 95°C 30 s, 58°C 30 s, and 72°C 1 min, with a final extension step at 72°C for 5 min. Four primer sets 16S 27f/16S907r (16SrRNA) (28), Tb11/Tb12 (*hsp65*) (Telenti et al., 1993), Myco-F/Myco-R (*rpoB*) (Adekambi and Drancourt, 2004), and 16S-1511f/23S-23r (ITS) (Harmsen et al., 2003) were used as control.

**TABLE 1:** Strains and the amplification in the actual PCR tests.

**Classification**	**Species**	**Strains**	**Gene targets for primers**						
			
			**Number**	**MTBC-ku**	**NTM-ku**	**16SrRNA**	***hsp65***	***rpoB***	**ITS**
MTBC standard strains	*M. tuberculosis*	H37Rv	1	+	−	+	+	+	+
	*M. bovis*	93006	1	+	−	+	+	+	+
NTM standard strains	*M. abscessus*	95021	1	−	+	+	+	+	+
	*M. aichiense*	95026	1	−	+	+	+	+	+
	*M. avium*	25291	1	−	+	+	+	+	+
	*M. bolletii*	95067	1	−	+	+	+	+	+
	*M. branderi*	95068	1	−	+	+	+	+	+
	*M. celatum*	95072	1	−	+	+	+	+	+
	*M. chelonae* subsp. *chelonae*	93419	1	−	+	+	+	+	+
	*M. diernhoferi*	95149	1	−	+	+	+	+	+
	*M. doricum*	95078	1	−	+	+	+	+	+
	*M. farcinogenes*	93487	1	−	+	+	+	+	+
	*M. farcinogenes*	95012	1	−	+	+	+	+	+
	*M. farcinogenes* subsp. *senegalense*	93488	1	−	+	+	+	+	+
	*M. flavescens*	95030	1	−	+	+	+	+	+
	*M. fluoranthenivorans*	95081	1	−	+	+	+	+	+
	*M. fortuitum* subsp. *fortuitum*	93555	1	−	+	+	+	+	+
	*M. fortuitum* subsp. *fortuitum*	93556	1	−	+	+	+	+	+
	*M. fortuitum* subsp. *fortuitum doricum*	93407	1	−	+	+	+	+	+
	*M. fortuitum* subsp. *fortuitum duvalii*	93396	1	−	+	+	+	+	+
	*M. frederiksbergense*	95083	1	−	+	+	+	+	+
	*M. gastri*	95006	1	−	+	+	+	+	+
	*M. gordonae*	93409	1	−	+	+	+	+	+
	*M. hassiacum*	95085	1	−	+	+	+	+	+
	*M. houstonense*	95091	1	−	+	+	+	+	+
	*M. kansasii*	93410	1	−	+	+	+	+	+
	*M. kansasii*	95013	1	−	+	+	+	+	+
	*M. komossense*	95093	1	−	+	+	+	+	+
	*M. kubicae*	95094	1	−	+	+	+	+	+
	*M. kumamotonense*	95095	1	−	+	+	+	+	+
	*M. lentiflavum*	95097	1	−	+	+	+	+	+
	*M. malmoense*	95100	1	−	+	+	+	+	+
	*M. malmoense*	95148	1	−	+	+	+	+	+
	*M. murale*	95143	1	−	+	+	+	+	+
	*M. nebraskense*	95105	1	−	+	+	+	+	+
	*M. neworleansense*	95106	1	−	+	+	+	+	+
	*M. palustre*	95109	1	−	+	+	+	+	+
	*M. peregrinum*	95131	1	−	+	+	+	+	+
	*M. saskatchewanense*	95117	1	−	+	+	+	+	+
	*M. senuense*	95118	1	−	+	+	+	+	+
	*M. smcgmatis*	Mc2^155^	1	−	+	+	+	+	+
	*M. sphagni*	95123	1	−	+	+	+	+	+
	*M. terrae*	95005	1	−	+	+	+	+	+
	*M. vaccae*	95003	1	−	+	+	+	+	+
	*M. intracellulare*	93519	1	−	+	+	+	+	+
	*M. marseillense*	95101	1	−	+	+	+	+	+
Clinical isolates	*M. tuberculosis*		42	+	−	+	+	+	+
	*M. abscessus*		10	−	+	+	+	+	+
	*M. marseillense*		10	−	+	+	+	+	+
	*M. avium*		10	−	+	+	+	+	+
	*M. kansasii*		10	−	+	+	+	+	+
Common respiratory pathogenic bacteria	*Corynebacterium Diphtheria*	CMCC38105	1	−	−	+	−	−	+
	*Klebsiella pneumoniae*	KP3093	1	−	−	+	−	−	+
	*Streptococcus pneumoniae*	ATCC49619	1	−	−	+	−	−	+
	*Mycoplasma pneumonia*	29342	1	−	−	+	−	−	+
	*Mycoplasma pneumonia*	MP39505	1	−	−	+	−	−	+
	*Staphylococcus aureus*	CICC21601	1	−	−	+	−	−	+
	*Staphylococcus aureus*	CO WanI	1	−	−	+	−	−	+
	*Haemophilus influenzae*	M5216 Hib	1	−	−	+	−	−	+
	*Neisseria meningitidis*	341201	1	−	−	+	−	−	+
	*Streptococcus pyogenes*	CICC10373	1	−	−	+	−	−	+
	*Legionella pneumophila*	ATCC33152	1	−	−	+	−	−	+
	*Legionella pneumophila*	9797	1	−	−	+	−	−	+
	*Legionella pneumophila*	9134	1	−	−	+	−	−	+
	*Nocardia farcinica*	12	1	−	−	+	+	+	+
	*Nocardia africana*	75	1	−	−	+	+	+	+
	*Nocardia farcinica*	14	1	−	−	+	+	+	+
	*Nocardia veterana*	81	1	−	−	+	+	+	+
	*Nocardia africana*	72	1	−	−	+	+	+	+
	*Nocardia nova*	86	1	−	−	+	+	+	+
	*Nocardia farcinica*	90	1	−	−	+	+	+	+
	*Nocardia veterana*	84	1	−	−	+	+	+	+
	*Nocardia paucivorans*	79	1	−	−	+	+	+	+
	*Nocardia abscessus*	71	1	−	−	+	+	+	+

### Statistical Analysis

Fourfold table Chi-square test was used to test the differences in variant rates between the *ku* and *rpoB* genes.

## Results

### Distribution and Polymorphism of the *ku* Gene in *Mycobacterium*

The *ku* gene was found to be distributed in almost all of the 7294 *Mycobacterium* genomes with three exceptions: two incomplete genomes, *M. setense* strain 852014-10208_SCH5295773 and *M. tuberculosis* strain 0109V, without sequence analogous to the *ku* gene, and an incomplete *ku* gene in *M. tuberculosis* strain AH26_28866, with the first 290 bp in contig NZ_LKMH01000091.1 and 304–822 bp in contig NZ_LKMH01000168.1.

In *M. tuberculosis*, the *ku* gene was highly conserved. Of the 5243 *M. tuberculosis* genomes, there were 39 *ku* genotypes, of which 5149 (98.17%) genomes harbored the Rv0937c genotype, while 25 genomes were one genome with one genotype (Supplementary Table 1). The similarity of the *ku* gene sequence of the 39 genotypes was also very high. Only 37 sites on the 822 bp of the *ku* gene had variants, and sites 287, 449, and 451 had the highest rate of variation, at 5.13% (2/39).

In MTBC, except for *M. tuberculosis*, 116 genomes of four species (*M. africanum*, *M. bovis*, *M. caprae*, *M. microti*, and *M. pinnipedii*) and five variants (*M. canettii*, *M. decipiens*, *M. mungi*, and *M. orygis*) had 14 genotypes and 17 variant sites, among which the sequences of RN09_1148 genotype of *M. africanum* (28/29), LH58_05105 of *M. bovis* (68/70), BBG46_05065 of *M. caprae* (2/2), MORY_05401 of *M. orygis* (1/1), MPS_4136 of *M. pinnipedii* (2/2), B8W66_10645 of *M. decipiens* (1/1) and RN08_1045 of *M. microti* (1/1) were completely identical to that of the Rv0937c genotype, as a result of which Rv0937c was the dominant genotype in the MTBC (98%, 5253/5359). On the other hand, nine *M. canettii* genomes were found to have six genotypes and two variant sites, A210G and A487G, which appeared only in all *M. canettii*, indicating obvious species specificity. In addition, from the high conservation of the *ku* gene in MTBC, we inferred that *M. tuberculosis* strain 0109V and *M. setense* strain 852014-10208_SCH5295773 might also carry the *ku* gene, and the missing of the *ku* gene was caused by the incompletion of the genomic data.

In NTM, 278 sites of the *ku* gene were conserved in all NTM genotypes, but the conservation of the *ku* gene in each species varied greatly (Supplementary Table 1). In some species, the *ku* gene was highly conserved and had few genotypes. For example, 91.4% (139/152) of *M. avium* were concentrated in three types (MAV_1050, IQU_02120, and O982_17680), 94.1% (16/17) of *M. immunogenum* in ABG82_05475, and 61.9% (13/21) of *M. kansasii* in MKSMC1_52900. However, most species are genotype polymorphic. There were nine genotypes among 10 genomes in *M. asiaticum* and 14 genotypes among 42 genomes in *M. chelonae*. Furthermore, each genome had a genotype in some species, such as *M. heckeshornense*, *M. llatzerense*, and *M. mageritense*.

**FIGURE 1:**
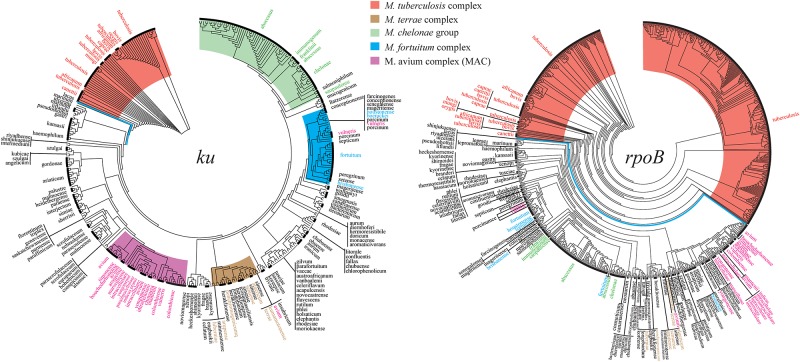
Phylogenetic trees drawn on the basis of the *ku* and *rpoB* genes. The *Mycobacterium tuberculosis* complex (MTBC) and the four complex groups of non-tuberculous mycobacteria (NTM) are highlighted by different colors.

Overall, 32.4% sites (266/822) of the *ku* gene were conserved in all *Mycobacterium* genotypes, but none of the NTM genotype were 100% identical to the genotypes of MTBC, including Rv0937c. On phylogeny tree based on the *ku* gene, MTBC could be clearly separated from NTM without any exception (Figure 1). The *ku* genotypes in different species but having identical sequences are listed in Table 2. For MTBC, only *M. canettii*, *M. decipiens*, and *M. mungi* had completely special genotypes, other species of MTBC could not be separated by the *ku* gene sequence. For NTM, the genotypes of *M. avium* and *M. intracellulare*, *M. abscessus*, and *M. chelonae* only accounted for a very small proportion. Therefore, except the other 18 NTM species in Table 2, most of the NTMs could be identified to species level by the *ku* gene sequence.

**TABLE 2:** *ku* genotypes in different species but with identical sequences.

	**Strain numbers**		**Genotypes**						
		
	**Genotypes**	**Species**							
	**involved**	**involved**							
MTBC	5250	5347	*M. africanum* CP010334	*M. bovis* NZ_CP009449	*M. capra*e NZ_CP016401	*M. microti* CP010333	*M. orygis* NZ_APKD01000011	*M. pinnipedii* NZ_PYQH00000000	*M. tuberculosis* NC_000962
NTM	2	9	*M. porcinum* NZ_MVIG01000003	*M. vulneris* NZ_CCBG010000002					
	2	8	*M. malmoense* NZ_MBEB01000153	*M. parascrofulaceum* NZ_GG770554					
	5	17	*M. indicuspranii* NC_018612	*M. intracellulare* NC_016948	*M. paraintracellulare* NZ_NCXN01000007	*M. yongonense* CP003347			
	2	4	*M. heckeshornense* NZ_MPJF01000011	*M. xenopi* NZ_AJFI01000095					
	4	15	*M. conceptionense* NZ_LFOD01000002	*M. mageritense* NZ_AGSZ01000485	*M. senegalense* NZ_LDCO01000015				
	10	20	*M. chimaera* NZ_CP012885	*M. intracellulare* NZ_JAON01000035					
	2	2	*M. austroafricanum* NZ_HG964452	*M. vanbaalenii* NC_008726					
	86	153	*M. avium* NC_008595	*M. bouchedurhonense* NZ_MVHL01000009					
	4	163	*M. avium* NZ_JAOD01000007	*M. intracellulare* NC_016946					
	6	1428	*M. abscessus* NZ_JMIA01000002	*M. chelonae* NZ_MAEQ01000007					

### Comparison of the *ku* Gene With the IS*6110* Element and the *rpoB* Gene

The distribution of the two most used identification loci, IS*6110* and *rpoB*, in the whole *Mycobacterium* genus had also been analyzed in this study.

The IS*6110* element has 16 completely identical copies in the *M. tuberculosis* strain H37Rv genome. There was at least one IS*6110* copy in 4450 *M. tuberculosis* genomes with identical sequence to the IS*6110* element of H37Rv. The IS*6110* element in 428 genomes were only partially identical to that of H37Rv. The IS*6110* of 306 *M. tuberculosis* genomes were located at the end of contig and were incomplete. The remaining 61 genomes, including the complete genome of *M. tuberculosis* UT205, had no sequences homologous to that of IS*6110*. In the other MTBC species/variants, the complete genomes of *M. canettii* CIPT 140070008 and 140070017 also had no sequences similar to that of IS*6110*. Therefore, even though it has been used as an important diagnostic marker to identify MTBC species (Coros et al., 2008; Guernier et al., 2017), the IS*6110* element was not common to all the MTBC strains, even *M. tuberculosis*. Tests based on it have false negatives, which is consistent with previous studies (Viana-Niero et al., 2006; Freidlin et al., 2017).

The *rpoB* gene was widely distributed in *Mycobacterium* and had a total of 861 genotypes, excluding 57 incomplete *rpoB* sequences from analysis (Supplementary Table 2). The *rpoB* gene had 454 variant sites in *M. tuberculosis* alone, compared to only 37 variant sites of the *ku* gene in *M. tuberculosis*. Even after standardization by gene length, the variant rate of *rpoB* gene in *M. tuberculosis* was 14.4% (454/3519 bp), which was far greater than that of the *ku* gene at 4.5% (37/822 bp) (Σ^2^ = 46.872, *P* < 0.01). The *rpoB* gene could set apart the MTBC from all NTM without any exception on its phylogenetic tree, but the four complex groups of NTM could not be separated as distinctly as the tree drawn on the *ku* gene (Figure 1).

### Scanning Similarities of the *ku* Gene in the Genomes of Other Families in *Corynebacteriales*

To confirm the results of Weller et al. (2002), we downloaded 14 whole genomic sequences of other families belonging to *Corynebacteriales* with *Mycobacteriaceae*, *Corynebacteriaceae* (CP008913.1, CP017639.1, CP026947.1, and CP026948.1), *Dietziaceae* (CP027238.1, and CP024869.1), *Gordoniaceae* (CP002907.1, NZ_CP025435.1, and CP023405.1), *Segniliparaceae* (CP001958.1), *Tsukamurellaceae* (CP001966.1), and *Nocardiaceae* (CP018082.1, CP032568.1, and CP016819.1) and blasted Rv0937c (NC_000962) with these genomes. The results showed that there was no region homologous to Rv0937c in these genomes.

### Development of PCR System for Identifying of MTBC and NTM

For the rarity of the *ku* gene in bacteria and the distinct clusters of MTBC and NTM on the phylogenetic tree, we inferred that mycobacteria might be specially identified from other bacteria and MTBC from NTM only by PCR without sequencing. Afterward, the PCR system was developed.

**FIGURE 2:**
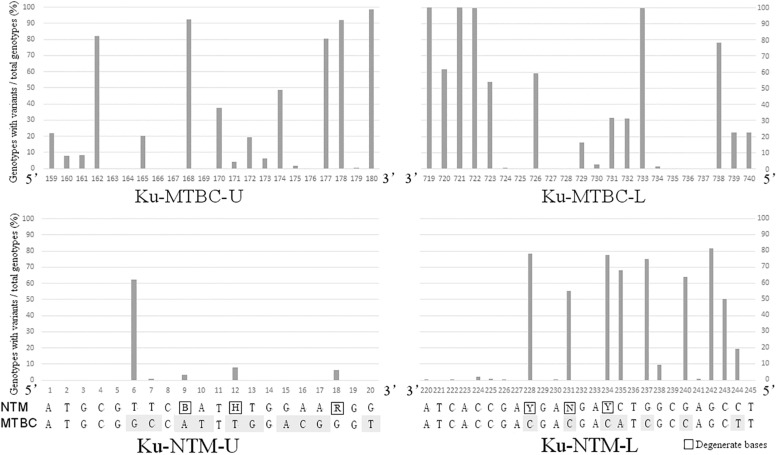
The variation rates of the primer design sites in NTM *ku* genotypes.

*Mycobacterium tuberculosis* complex-specific primers were designed in the conservative regions that were identical in the 55 *ku* genotypes of MTBC, but different to all *ku* genotypes of NTM. Candidate primer sets with different product lengths were designed and screened. The pair with the most sufficient difference between MTBC and NTM was selected. ku-MTBC-U: GGT GGT CGA CTA CCG CGA TCT T and ku-MTBC-L: TCT TCG GGC TCG TCC AGC AAC C were located at 159–180 bp and 719–740 bp of the reference sequence of Rv0937c genotype, respectively. Figure 2 shows that the design region of this pair of primers has a high degree of variation in NTM. Especially, the first base of the 3′ end of the upstream primer and the first, third and fourth base of the 3′ end of the downstream primer were single nucleotide polymorphic loci that completely distinguished MTBC from NTM. Simulated PCR revealed that 55 MTBC genotypes could be amplified by this primer set, but 324 NTM genotypes could not be amplified.

NZ_CP009616.seq of *M. abscessus* was used as reference of NTM for primer design. By marking the completely conserved sites in NTM on the reference sequence, it was found that the initial 1–20 bp of *ku* gene was the best region for the design of upstream primer ku-NTM-U with the most conserved sites in NTM and nine base difference to MTBC (Figure 2). The downstream primer Ku-NTM-L was an NTM/MTBC universal primer at 220–245 bp with eight conserved bases at the 3′ end. To improve the amplification efficiency of all NTMs, degenerate base were used. The primer set was ku-NTM-U: ATG CGT TCB ATH TGG AAR GG and ku-NTM-L: AGG CTC GCC AGR TCN TCR TCG GTG AT, and its specificity for NTM depends on the upstream primer.

In the actual PCR amplification of the 148 *Mycobacterium* and common respiratory pathogenic bacteria, all of the MTBC strains were positive for the amplification of ku-MTBC-U/L and negative for ku-NTM-U/L. The amplifications of NTM strains were opposite, and all of the 23 respiratory pathogenic bacteria were negative for both of them. The sensitivity and specificity of the two pairs of primers were 100%. Among the control primers, primers of 16SrRNA and ITS were universal and almost all of the tested strains, including the 23 respiratory pathogenic strains, were positive for them. Primers for *hsp60* and *rpoB* were specific for *Mycobacterium*. But all of the 10 *Nocardia* strains belonging to six *Nocardia* species were positive, although other respiratory pathogenic strains were negative (Table 1). Without sequencing, none of the four pairs of primers would be suitable for the identification of *Mycobacterium*, let alone the distinction between MTBC and NTM.

## Discussion

The GenBank database contains plenty of *Mycobacterium* genome data. The sensitivity and specificity of molecular detection methods can be predicted and compared before actual use. In this work, approximately 151 definite species/variants of *Mycobacterium* have been included, accounting for 79.7% of all known mycobacterial species and all submitted genomes. This work has the same coverage as the phylogenomics and comparative genomics studies of Gupta et al. (2018). Although it does not cover all the *Mycobacterium* species, it may be the most comprehensive analysis that can be done so far and includes all of the clinically common species.

The similarity between the eukaryotic and bacterial Ku proteins suggested that they were evolved from a common ancestor and very ancient process (Weller et al., 2002). The *ku* gene should be present in the *Mycobacterium* genome before the differentiation of *Mycobacterium* species. Additionally, The Ku-based NHEJ system participates in the repairing of DSBs of *Mycobacterium*, which maintains the genome integrity and is pivotal for cell survival. The *ku* gene is less likely to be lost in the evolution of *Mycobacterium*. Thus, it is reasonable that the *ku* gene is distributed and conserved in all sequenced *Mycobacterium* genomes. Moreover, the research of Weller et al. (2002) has speculated that the Ku ligase system might be related to the special growth stage of mycobacteria, especially the bacterial sporulation and the long stationary phase of life cycle. Different growth rates and culture and biochemical test characteristics are just important indicators for distinguishing *Mycobacterium* species, especially NTM. Therefore, in theory, it is not unexpected that the *ku* gene can completely distinguish MTBC from NTM and distinguish species in NTM.

Among the detection methods of *Mycobacterium*, acid-fast (AF) staining, also known as Ziehl–Neelsen stain, is currently the most widely used preliminary diagnostic method. The sensitivity of AF staining compared with culture ranges from 22 to 78%, and its limit of detection ranges from 5 × 10^3^ to 1 × 10^4^ bacilli/mL. However, AF staining is not specific for *Mycobacterium* detection, and *Mycobacterium* spp. cannot be distinguished from other AF bacteria, such as *Nocardia*, *Rhodococcus*, *Tsukamurella*, *Gordona*, *Dietzia*, *Legionella micdadei*, *Cryptosporidium*, *Isospora belli*, and *Cyclospora cayetanensis* and parasites such as *Sarcocystis* and *Taenia saginata*. Moreover, non-AF tuberculosis bacilli (Vilcheze and Kremer, 2017) also exist.

Thus, nucleic acid detection methods (NADMs) are becoming more and more important in the diagnosis and identification of mycobacteria (Niemann et al., 2000; Brunello et al., 2001). Loci of developed NADM include species-specific insertion sequences, such as IS*6110* for members of the *M. tuberculosis* complex (Thierry et al., 1990), IS*900* and *F57* for *M. avium* subsp. *paratuberculosis* (Slana et al., 2008), IS*901* for *M. avium* subsp. *avium* (Slana et al., 2010), IS*2404* and IS*2606* for *M. ulcerans, M. liflandii*, *M. pseudoshottsii*, and *M. shottsii* (mycolactone-producing mycobacteria) (Fyfe et al., 2007), and common shared bacterial genes, for example *16S rRNA*, *hsp65*, *rpoB* genes and the internal transcribed spacer (ITS) of broad-range sequencing approaches. Multi-genes analysis (Homolka et al., 2012; Perez-Lago et al., 2014; Gupta et al., 2018) and whole genome sequencing (Vissa et al., 2009; Fedrizzi et al., 2017; Trofimov et al., 2018) have also been used in *Mycobacterium* and because of their high resolution, they can identify mycobacteria to the species level. Methods based on sequencing and homology comparison, especially on sequences of hundreds of core genes or whole genome are more available for research purposes, and they are promising tools in the identification of new mycobacteria species, new molecules for bacterial typing and new candidate genes for multidrug resistance. But they are less practical for rapid screening of large samples and for primary hospitals without the ability of bioinformatics analysis. This is also the reason that single locus, IS*6110* and *rpoB*, are the most commonly used biomarkers. But IS*6110* cannot be applied to NTM. Although *rpoB* is used not only for identification but also for antibiotic resistance prediction, and GeneXpert assay which based on it requires little technical training and can obtained from unprocessed sputum samples in 90 min, with minimal biohazard. GeneXpert is replacing AF and endorsed by the World Health Organization (WHO) (Moure et al., 2011; Li et al., 2017), but it is also only applicable to MTBC and requires special instruments. In fact, until now, there has been no specific biomarker that is commonly used for all mycobacteria without sequencing.

The analysis in this study showed the value of the *ku* gene as a diagnostic biomarker. The *ku* gene is not a common gene of bacteria. Its rarity in prokaryotes (Weller et al., 2002), especially its absence in bacteria closely related to *Mycobacterium* (such as *Nocardia*), endows it with high specificity. Its wide distribution in all sequenced *Mycobacterium* makes it widely applicable for MTBC and NTM. Both features actualize the greatest application value of the *ku* gene, that is, they can directly distinguish mycobacteria, MTBC and NTM by PCR, and achieve the purpose of rapid clinical diagnosis. This actual application value has been confirmed by the MTBC/NTM- specific primers we designed and the testing of the standard and clinical strains. In conclusion, Ku gene is a new single-gene biomarker of *Mycobacterium* that differentiates MTBC and NTM and makes the identification of MTBC and NTM simpler and more accurate. However, it does not define the resistance of *Mycobacterium*, so it can’t take into account the identification of strains and the prediction of drug resistance simultaneously as *ropB* gene does. Its application value lies in that it can be used as a single indicator for the primary screening and identification of mycobacteria, or can be combined with *rpoB* to complement its deficiencies for accurate identification of *Mycobacterium*. More sensitive detection methods based on this gene and application for the detection of different samples besides pure cultures will be explored in our further study to help the diagnosis of Tb worldwide.

## Data Availability

All datasets generated for this study are included in the manuscript and/or the Supplementary Files.

## Author Contributions

RW conceived the project, analyzed and interpreted the data, designed the primers, and prepared the figures and manuscript. LZ, CM, and TX did the PCR testing. ML and XZ collected the strains. HL downloaded the *Mycobacterium* genomes from the GenBank database. KW revised the manuscript. All authors had full access to all the data in the study and approved the final version of the manuscript for submission.

## Conflict of Interest Statement

The authors have applied for a pending Chinese patent titled “The application of the Ku protein in *Mycobacterium*,” Number: 201910154948.5.
