# Supplementary material for: A New Single Gene Differential Biomarker for Mycobacterium tuberculosis Complex and Non-tuberculosis Mycobacteria
Source: Front Microbiol. 2019 Aug 13;10:1887. doi: 10.3389/fmicb.2019.01887 (PMC6700215; doi:10.3389/fmicb.2019.01887)
Supplement: Supplementary file 1 [file Data_Sheet_1.PDF]

Supplementary table 1. Representative sequences of the *ku* genotypes in each *Mycobacterium* species/variants.

Numbers	Species/variants	Accession	Locus tag	Amino acids	Beginning of position	End of position	Strain number with this genotype	Total genotype number	Total genome number
1	*M. abscessus*	NZ_FVYB01000028.1	B5072_RS24850	269	5117	5926	470		
2	*M. abscessus*	NZ_CP009616.1	NF90_18295	269	3724150	3724959	258		
3	*M. abscessus*	NZ_FSOT01000007.1	BUW87_RS17885	269	5134	5943	146		
4	*M. abscessus*	NZ_FSOJ01000010.1	BUU53_RS20280	269	19476	20285	62		
5	*M. abscessus*	NZ_FSQK01000008.1	BUV02_RS10600	269	5138	5947	56		
6	*M. abscessus*	NZ_AKUA01000005.1	MA5S0708_2077	269	5088	5897	55		
7	*M. abscessus*	NZ_CP014957.1	A3O02_05330	269	1080403	1081212	49		
8	*M. abscessus*	NZ_FVUH01000007.1	B5322_RS21745	269	105165	105974	37		
9	*M. abscessus*	NZ_FVXW01000006.1	B5471_RS09480	269	5133	5942	31		
10	*M. abscessus*	NZ_FVRY01000004.1	B5Y99_RS21495	269	121133	121942	23		
11	*M. abscessus*	NZ_FSEW01000004.1	BUW78_RS09630	269	106044	106853	21		
12	*M. abscessus*	NZ_FVDA01000001.1	B5519_RS01630	269	327157	327966	21		
13	*M. abscessus*	NZ_FVXY01000006.1	B5268_RS16860	269	5132	5941	20		
14	*M. abscessus*	NZ_CSUB01000008.1	AKY83_RS21895	269	94851	95660	13		
15	*M. abscessus*	NZ_FVWN01000016.1	B5382_RS23185	269	5136	5945	13		
16	*M. abscessus*	NZ_FVJY01000005.1	B5204_RS23635	269	375873	376682	10		
17	*M. abscessus*	NZ_AHAS01000006.1	MBOL_RS18050	269	84145	84954	9		
18	*M. abscessus*	NZ_FVBG01000001.1	B5408_RS03615	269	711085	711894	9		
19	*M. abscessus*	NZ_FSBV01000005.1	BUV30_RS10475	269	47054	47863	8		
20	*M. abscessus*	NZ_FVYW01000004.1	B5323_RS16835	269	443794	444603	8		
21	*M. abscessus*	NZ_FSCN01000005.1	BUV47_RS15315	269	324747	325556	7		
22	*M. abscessus*	NZ_FRZG01000004.1	BUT85_RS13140	269	373287	374096	4		
23	*M. abscessus*	NZ_FSKA01000001.1	BUV41_RS12785	269	2499339	2500148	4		
24	*M. abscessus*	NZ_FVCU01000001.1	B5116_RS05140	269	1040867	1041676	4		
25	*M. abscessus*	NZ_FVNM01000004.1	B5203_RS14890	269	438309	439118	4	50	1376
26	*M. abscessus*	NZ_CP014950.1	A3N95_04720	269	953230	954039	3		
27	*M. abscessus*	NZ_FSFR01000002.1	BUS48_RS12890	269	292086	292895	2		
28	*M. abscessus*	NZ_FSJJ01000003.1	BUT72_RS05210	269	6817	7626	2		
29	*M. abscessus*	NZ_FSPU01000007.1	BUU77_RS15545	269	5157	5966	2		
30	*M. abscessus*	NZ_FVDQ01000001.1	B5296_RS05540	269	1116933	1117742	2		
31	*M. abscessus*	NZ_FVMQ01000016.1	B5Z11_RS24400	269	28489	29298	2		
32	*M. abscessus*	NZ_FVNO01000005.1	SAMEA2070714_03418	269	62883	63692	2		
33	*M. abscessus*	NZ_FVVE01000004.1	B5Y72_RS12945	269	317747	318556	2		
34	*M. abscessus*	NC_021282.1	MASS_RS05400	269	1068765	1069574	1		
35	*M. abscessus*	NZ_AJLY02000015.1	UUG_RS05820	269	99890	100699	1		
36	*M. abscessus*	NZ_AJLZ01000003.1	UUM_RS0107755	269	257859	258668	1		
37	*M. abscessus*	NZ_CP012044.1	MAUC22_RS05900	269	1183422	1184231	1		
38	*M. abscessus*	NZ_FSAN01000003.1	BUV59_RS15685	269	33361	34170	1		
39	*M. abscessus*	NZ_FSCC01000006.1	BUT88_RS18635	269	244449	245258	1		

40	*M. abscessus*	NZ_FSLU01000002.1	SAMEA2275812_01380	269	384284	385093	1		
41	*M. abscessus*	NZ_FVFY01000002.1	B5149_RS09755	269	445292	446101	1		
42	*M. abscessus*	NZ_FVLE01000005.1	B5152_RS19150	269	457611	458420	1		
43	*M. abscessus*	NZ_FVNF01000013.1	B5Y47_RS23030	269	93803	94612	1		
44	*M. abscessus*	NZ_FVPZ01000001.1	B5168_RS02145	269	421984	422793	1		
45	*M. abscessus*	NZ_FVTC01000010.1	B5062_RS20040	269	93813	94622	1		
46	*M. abscessus*	NZ_FVVK01000002.1	B5046_RS12915	269	831980	832789	1		
47	*M. abscessus*	NZ_FVXU01000019.1	B5432_RS22280	269	50298	51107	1		
48	*M. abscessus*	NZ_FWCU01000005.1	B6F47_RS23485	269	326078	326887	1		
49	*M. abscessus*	NZ_HE817967.1	MABCF_RS18555	269	1051369	1052178	1		
50	*M. abscessus*	NZ_JMIA01000002.1	EG21_RS0105785	269	330101	330910	1		
51	*M. acapulcensis*	NZ_LT592223.1	BN8058_RS00740	318	143700	144656	1	1	1
52	*M. africanum*	CP010334.1	RN09_1148	273	1042099	1042920	28	2	29
53	*M. africanum*	NZ_KK338758.1	CH83_00973	273	1042089	1042910	1		
54	*M. algericum*	NZ_MVHC01000005.1	BST10_07700	296	232191	233081	1	1	1
55	*M. alsense*	NZ_MVHD01000046.1	BST11_20930	310	21673	22605	1	1	1
56	*M. angelicum*	NZ_MVHE01000038.1	BST12_19390	305	8582	9499	1	1	1
57	*M. aromaticivorans*	NZ_JALN02000001.1	Y900_008515	305	1738320	1739237	1	1	1
58	*M. arosiense*	NZ_MVHG01000014.1	BST14_08735	300	31985	32887	1	1	1
59	*M. arupense*	NZ_MVHH01000037.1	BST15_15410	288	3359	4225	2	1	2
60	*M. asiaticum*	NZ_MVHI01000005.1	BST16_05335	310	116755	117687	2	9	10
61	*M. asiaticum*	NZ_LZKQ01000296.1	A9X01_04795	306	6391	7311	1		
62	*M. asiaticum*	NZ_LZKS01000175.1	A5661_10270	310	13270	14202	1		
63	*M. asiaticum*	NZ_LZLF01000166.1	A9W94_17300	310	2284	3216	1		
64	*M. asiaticum*	NZ_LZLM01000109.1	A5640_20875	310	7029	7961	1		
65	*M. asiaticum*	NZ_LZLQ01000136.1	A5636_13390	316	22708	23658	1		
66	*M. asiaticum*	NZ_LZLR01000123.1	A5635_24415	310	8543	9475	1		
67	*M. asiaticum*	NZ_LZLS01000091.1	A5634_22185	268	11	817	1		
68	*M. asiaticum*	NZ_LZMH01000037.1	A5645_11040	310	33809	34741	1		
69	*M. aurum*	NZ_CVQQ01000008.1	AURUM_RS14190	300	141313	142215	1	2	2
70	*M. aurum*	NZ_LT549889.1	BN4366_RS18130	310	3825250	3826182	1		
71	*M. austroafricanum*	NZ_HG964452.1	BN976_RS13145	306	567884	568804	1	1	1
72	*M. avium*	NC_008595.1	MAV_1050	302	986287	987195	85	11	152
73	*M. avium*	NZ_AGAR01000223.1	IQU_02120	302	654	1562	33		
74	*M. avium*	NZ_AYNT01000186.1	O982_17680	302	8389	9297	21		
75	*M. avium*	NZ_AFNS01000734.1	MAPCLIJ361_20635	302	8413	9321	3		
76	*M. avium*	NZ_BDMZ01000035.1	B9B21_RS09430	302	9078	9986	3		
77	*M. avium*	NZ_AYNQ01000562.1	O971_16840	302	8309	9217	2		
78	*M. avium*	NC_002944.2	MAP0875c	302	898252	899160	1		
79	*M. avium*	NZ_AGAQ01000322.1	KEG_RS011364	291	4833	5708	1		
80	*M. avium*	NZ_BDOC01000060.1	B9C11_RS12035	302	34779	35687	1		
81	*M. avium*	NZ_JAOD01000007.1	I548_3734	309	620126	621055	1		
82	*M. avium*	NZ_LNBR01000001.1	A4U18_RS00280	302	64157	65065	1		

83	*M. bacteremicum*	NZ_MVHJ01000008.1	BST17_RS12055	313	146847	147788	1	1	1
84	*M. boenickei*	NZ_FUWC01000001.1	B2D00_RS13250	292	2740151	2741029	1	1	1
85	*M. bohemicum*	NZ_LQOK01000050.1	AWB93_00355	307	8637	9560	1	1	1
86	*M. bouchedurhonense*	NZ_MVHL01000009.1	BST19_08730	302	13179	14087	1	1	1
87	*M. bovis*	NZ_CP009449.1	LH58_05105	273	1039086	1039907	68	3	70
88	*M. bovis*	NZ_AWPL01000022.1	O217_05120	273	75481	76302	1		
89	*M. bovis*	NZ_KK308915.1	Z584_RS16600	273	20334	21155	1		
90	*M. branderi*	NZ_MVHM01000004.1	BST20_10495	306	137905	138825	1	1	1
91	*M. brisbanense*	NZ_BCSX01000007.1	RMCB_0550	312	66787	67725	1	1	1
92	*M. canariasense*	NZ_BCSY01000087.1	RMCC_5622	302	51767	52675	2	1	2
93	*M. canettii*	NZ_CAOM01000084.1	QZ53_RS05005	273	30639	31460	3	6	9
94	*M. canettii*	NZ_CAOL01000103.1	QZ38_RS05080	273	77842	78663	2		
95	*M. canettii*	NC_015848.1	MCAN_09371	273	1050440	1051261	1		
96	*M. canettii*	NC_019951.1	BN42_20747	273	1081896	1082717	1		
97	*M. canettii*	NC_019952.1	BN45_20238	273	1091345	1092166	1		
98	*M. canettii*	NZ_CAON01000097.1	QZ52_RS05040	273	18282	19103	1		
99	*M. caprae*	NZ_CP016401.1	BBG46_05065	273	1068498	1069319	2	1	2
100	*M. celatum*	NZ_BBUN01000221.1	AWU83_RS13460	297	1430	2323	2	1	2
101	*M. celeriflavum*	NZ_MVHN01000010.1	BST21_11750	312	115150	116088	1	1	1
102	*M. chelonae*	NZ_MAFA01000017.1	B4402_RS08030	269	14795	15604	18	14	42
103	*M. chelonae*	NZ_MAEQ01000007.1	B4397_RS05725	269	54140	54949	5		
104	*M. chelonae*	NZ_LQON01000001.1	AWB96_00250	269	53874	54683	4		
105	*M. chelonae*	NZ_MAES01000025.1	B4392_RS22195	269	57994	58803	2		
106	*M. chelonae*	NZ_MLIJ01000019.1	BKG75_23920	269	1191620	1192429	2		
107	*M. chelonae*	NZ_MLIQ01000011.1	BKG82_07740	269	1119555	1120364	2		
108	*M. chelonae*	NZ_MLIT01000010.1	BKG85_06165	269	8471	9280	2		
109	*M. chelonae*	NZ_MAER01000004.1	B4381_RS00645	269	15415	16224	1		
110	*M. chelonae*	NZ_MAEU01000003.1	B4395_RS01770	269	209888	210697	1		
111	*M. chelonae*	NZ_MLID01000009.1	BKG69_06425	269	556736	557545	1		
112	*M. chelonae*	NZ_MLIL01000002.1	BKG77_10945	269	1377661	1378470	1		
113	*M. chelonae*	NZ_MLIO01000015.1	BKG80_18305	269	309215	310024	1		
114	*M. chelonae*	NZ_MLIP01000001.1	BKG81_05305	269	1115610	1116419	1		
115	*M. chelonae*	NZ_MLIU01000028.1	BKG86_13365	269	2624009	2624818	1		
116	*M. chimaera*	NZ_CP012885.1	AN480_29475	309	1185597	1186526	9	1	9
117	*M. chlorophenolicum*	NZ_BCQY01000016.1	MCH01S_RS21410	308	135498	136424	2	1	2
118	*M. chubuense*	NC_018027.1	Mycch_4293	303	4504040	4504951	1	2	3
119	*M. chubuense*	NZ_JYNX01000058.1	MCHUDSM44219_04094	308	271935	272861	2		
120	*M. colombiense*	NZ_LZKX01000184.1	A9W93_17450	301	7080	7985	4	12	17
121	*M. colombiense*	NZ_CP020821.1	B9D87_RS14325	301	3139443	3140348	2		
122	*M. colombiense*	NZ_LT719131.1	B0530_RS26775	301	1412802	1413707	1		
123	*M. colombiense*	NZ_LZJS01000128.1	A5685_08665	301	173767	174672	1		
124	*M. colombiense*	NZ_LZKI01000195.1	A5708_10825	301	64916	65821	1		
125	*M. colombiense*	NZ_LZKW01000132.1	A5623_13160	301	8273	9178	2		

126	*M. colombiense*	NZ_LZMA01000075.1	A5653_18405	301	24993	25898	1	12	17
127	*M. colombiense*	NZ_LZSX01000032.1	A5760_06090	301	190659	191564	1		
128	*M. colombiense*	NZ_MBEI01000061.1	A5732_20465	301	170149	171054	1		
129	*M. colombiense*	NZ_MBEN01000145.1	A5737_09820	301	62089	62994	1		
130	*M. colombiense*	NZ_MBEO01000119.1	A5738_13785	301	34791	35696	1		
131	*M. colombiense*	NZ_MBEP01000021.1	A5739_00870	301	197726	198631	1		
132	*M. conceptionense*	NZ_LZSK01000085.1	A5718_20395	294	17114	17998	5		
133	*M. conceptionense*	NZ_LZLN01000158.1	A5639_28845	294	36609	37493	3	4	11
134	*M. conceptionense*	NZ_MBGF01000133.1	A5743_19845	294	112136	113020	2		
135	*M. conceptionense*	NZ_LFOD01000002.1	ACT17_04690	294	299500	300384	1		
136	*M. confluentis*	NZ_LQOQ01000041.1	AWB99_24945	315	200192	201139	1	1	1
137	*M. conspicuum*	NZ_LQOR01000049.1	AWC00_22115	296	74555	75445	1	1	1
138	*M. cosmeticum*	NZ_CCBB010000001.1	BN977_01905	296	1961754	1962644	1	1	1
139	*M. diernhoferi*	NZ_MPNS01000025.1	BRW64_26560	300	28733	29635	2	1	2
140	*M. decipiens*	GCA_002104675.1	B8W66_10645	273	16705	17523	1	1	1
141	*M. doricum*	NZ_LQOS01000011.1	AWC01_02805	311	9751	10686	1	1	1
142	*M. elephantis*	NZ_LBNO01000001.1	AAV95_00340	312	69108	70046	2	1	2
143	*M. engbaekii*	NZ_LQOT01000030.1	AWC02_08820	292	91381	92259	1	1	1
144	*M. europaeum*	NZ_LQOU01000064.1	AWC03_24835	302	100832	101740	1	1	1
145	*M. fallax*	NZ_LQOJ01000048.1	AWC04_14755	268	48834	49640	1	1	1
146	*M. farcinogenes*	NZ_HG964482.1	BN975_04437	294	1717663	1718547	1	1	1
147	*M. flavescens*	NZ_MIHA01000017.1	BHQ18_21150	324	98502	99476	1	1	1
148	*M. florentinum*	NZ_LQOV01000003.1	AWC05_09425	291	204682	205557	1	1	1
149	*M. fortuitum*	NZ_LZSS01000144.1	A5755_18530	301	8058	8963	4		
150	*M. fortuitum*	NZ_LZIP01000103.1	A5768_06090	301	107496	108401	3		
151	*M. fortuitum*	NZ_LZIV01000116.1	A5670_08820	301	32627	33532	3		
152	*M. fortuitum*	NZ_BCSZ01000064.1	RMCFA_5969	301	224607	225512	1		
153	*M. fortuitum*	NZ_CP011269.1	XA26_13230	301	1367699	1368604	1		
154	*M. fortuitum*	NZ_JASW01000043.1	BI85_RS0118815	269	134440	135249	1		
155	*M. fortuitum*	NZ_LZIW01000116.1	A5669_02510	301	27639	28544	1	13	20
156	*M. fortuitum*	NZ_LZKO01000134.1	A5664_00585	301	76217	77122	1		
157	*M. fortuitum*	NZ_LZLO01000023.1	A5638_02240	301	235662	236567	1		
158	*M. fortuitum*	NZ_LZLP01000083.1	A5637_01720	301	241035	241940	1		
159	*M. fortuitum*	NZ_LZLZ01000011.1	A5654_09405	301	27383	28288	1		
160	*M. fortuitum*	NZ_MBEK01000074.1	A5734_29910	301	29472	30377	1		
161	*M. fortuitum*	NZ_MBER01000154.1	A5742_10670	301	6624	7529	1		
162	*M. fragae*	NZ_LQOW01000006.1	AWC06_09050	310	139116	140048	1	1	1
163	*M. franklinii*	NZ_MAFQ01000006.1	B4393_RS10100	269	53531	54340	2		
164	*M. franklinii*	NZ_MAFS01000007.1	B4388_RS08660	269	217395	218204	2	3	5
165	*M. franklinii*	NZ_MLIK01000004.1	BKG76_05030	269	917911	918720	1		
166	*M. gastri*	NZ_LQOX01000090.1	AWC07_05015	291	24712	25587	2	1	2
167	*M. genavense*	NZ_JAGZ01000004.1	T428_RS0111915	286	330576	331436	1	1	1
168	*M. gilvum*	NC_009338.1	Mflv_1826	328	1895574	1896560	1	2	2

169	*M. gilvum*	NC_014814.1	Mspyr1_12260	319	1247141	1248127	1	[illegible]	[illegible]
170	*M. goodii*	NZ_CP012150.1	AFA91_12775	316	2707452	2708402	1	1	1
171	*M. gordonae*	NZ_MIHG01000025.1	BHQ23_07820	308	5963	6889	2	5	6
172	*M. gordonae*	NZ_LKTM01000363.1	AO501_04605	313	14388	15329	1		
173	*M. gordonae*	NZ_LZLK01000165.1	A9W97_25780	318	20815	21771	1		
174	*M. gordonae*	NZ_LZLX01000331.1	A5656_06880	313	31076	32017	1		
175	*M. gordonae*	NZ_MAEM01000496.1	A9W98_33470	308	538	1464	1		
176	*M. haemophilum*	NZ_LDPQ01000005.1	ABH39_06585	285	70076	70933	4	2	5
177	*M. haemophilum*	NZ_CP011883.2	B586_06185	293	3449057	3449938	1		
178	*M. hassiacum*	NZ_AMRA01000097.1	C731_3536	318	17695	18651	2	1	1
179	*M. heckeshornense*	NZ_LFOF01000003.1	ACT16_03095	304	74356	75270	1	2	2
180	*M. heckeshornense*	NZ_MPJF01000011.1	BMW24_05110	309	10537	11466	1		
181	*M. heidelbergense*	NZ_MVHR01000051.1	BST25_21695	294	18693	19577	1	1	1
182	*M. heraklionense*	NZ_LDPO01000001.1	ABW16_02365	291	480459	481334	1	6	6
183	*M. heraklionense*	NZ_LZIU01000014.1	A5671_12590	293	4847	5728	1		
184	*M. heraklionense*	NZ_LZKB01000133.1	A5715_02990	297	3594	4487	1		
185	*M. heraklionense*	NZ_LZLB01000035.1	A5631_08640	293	4831	5712	1		
186	*M. heraklionense*	NZ_LZME01000025.1	A5649_15715	288	4477	5343	1		
187	*M. heraklionense*	NZ_MBEL01000117.1	A5735_16425	292	33766	34644	1		
188	*M. hiberniae*	NZ_LQOZ01000020.1	AWC09_08850	301	223329	224234	1	1	1
189	*M. holsaticum*	NZ_MIGZ01000018.1	BHQ17_04975	311	14502	15437	1	1	1
190	*M. houstonense*	NZ_LT546207.1	BN4332_RS15235	298	3020937	3021833	1	1	1
191	*M. icosiumassiliensis*	NZ_LT546166.1	BN4346_RS25975	293	486624	487505	1	1	1
192	*M. immunogenum*	NZ_CP011530.1	ABG82_05475	269	1101689	1102498	16	2	17
193	*M. immunogenum*	NZ_LQYE01000031.1	AWB85_15235	269	105681	106490	1		
194	*M. indicus pranii*	NC_018612.1	MIP_01541	309	1082961	1083890	1	1	1
195	*M. insubricum*	NZ_MVHS01000026.1	BST26_12015	267	16476	17279	1	1	1
196	*M. interjectum*	NZ_FJVQ01000140.1	BN4348_RS19850	306	68339	69259	1	2	2
197	*M. interjectum*	NZ_LQPB01000071.1	AWC11_01725	306	7280	8200	1		
198	*M. intermedium*	NZ_MVHT01000072.1	BST27_22015	298	25391	26287	3	1	3
199	*M. intracellulare*	NC_016946.1	OCU_09270	309	909953	910882	3	8	11
200	*M. intracellulare*	NZ_BAGQ01000088.1	MI198_RS15325	309	38812	39741	2		
201	*M. intracellulare*	NC_016948.1	OCQ_09360	309	923742	924671	1		
202	*M. intracellulare*	NZ_ABIN01000048.1	MIA_RS05470	309	9938	10867	1		
203	*M. intracellulare*	NZ_JAOM01000005.1	RT24_RS05375	258	221434	222210	1		
204	*M. intracellulare*	NZ_JAON01000035.1	L842_1073	309	242161	243090	1		
205	*M. intracellulare*	NZ_LZJO01000123.1	A5690_03855	309	24367	25296	1		
206	*M. intracellulare*	NZ_LZJT01000143.1	A5684_14260	309	21681	22610	1		
207	*M. iranicum*	NZ_AUWT01000034.1	N420_RS0117575	316	12639	13589	1	3	3
208	*M. iranicum*	NZ_LQPC01000015.1	AWC12_03840	316	62522	63472	1		
209	*M. iranicum*	NZ_LWCS01000023.1	A4X20_20560	317	75429	76382	1		
210	*M. kansasii*	NZ_JNDJ01000152.1	MKSMC1_52900	296	11333	12223	13		
211	*M. kansasii*	NZ_CP019883.1	B1T43_20020	296	4382751	4383641	1		

212	*M. kansasii*	NZ_JANZ01000004.1	I546_3192	296	373358	374248	2		
213	*M. kansasii*	NZ_LWCH01000070.1	A4G26_24955	301	2431	3336	1	8	21
214	*M. kansasii*	NZ_LWCI01000107.1	A4G28_08660	296	41625	42515	1		
215	*M. kansasii*	NZ_LWCK01000141.1	A4G29_08800	301	24261	25166	1		
216	*M. kansasii*	NZ_LWCM01000142.1	A4G31_08315	296	4582	5472	1		
217	*M. kansasii*	NZ_MVBN01000002.1	BZL29_2910	345	1026684	1027550	1		
218	*M. koreense*	NZ_NCXO01000015.1	B8W67_08910	297	58447	59340	1	1	1
219	*M. kubicae*	NZ_LQPD01000001.1	AWC13_00150	311	29089	30027	1	1	1
220	*M. kumamotonense*	NZ_LFOE01000001.1	ACT18_01630	291	286657	287532	2	1	2
221	*M. kyorinense*	NZ_BBKA01000018.1	TY31_RS03760	306	46061	46981	2		
222	*M. kyorinense*	NZ_LQPE01000085.1	AWC14_26805	306	16508	17428	1	3	4
223	*M. kyorinense*	NZ_LZKJ01000049.1	A5707_14820	306	55946	56866	1		
224	*M. lacus*	NZ_LQPF01000086.1	AWC15_21620	295	18307	19197	1	1	1
225	*M. leprae*	NC_011896.1	MLBR_RS10555	#VALUE!	2488109	2488862	4	1	4
226	*M. lepromatosis*	NZ_JRPY01000090.1	MLPM_RS10540	#VALUE!	7118	7974	1	1	1
227	*M. liflandii*	NC_020133.1	MULP_04792	291	5221008	5221883	1	1	1
228	*M. litorale*	NZ_CP019882.1	B1R94_23505	307	4950037	4950960	1	1	1
229	*M. llatzerense*	NZ_JXST01000001.1	TL10_RS00705	264	140569	141363	1	2	2
230	*M. llatzerense*	NZ_LIPZ01000016.1	BMI94_RS09490	264	32454	33248	1		
231	*M. longobardum*	NZ_LQPG01000008.1	AWC16_04595	288	72851	73717	1	1	1
232	*M. mageritense*	NZ_AGSZ01000485.1	MFOR_22840	294	2765	3649	1	2	2
233	*M. mageritense*	NZ_CCBF010000001.1	BN978_04259	304	4366810	4367724	1		
234	*M. malmoense*	NZ_MBEA01000230.1	A5674_05745	302	13088	13996	1		
235	*M. malmoense*	NZ_MBEB01000153.1	A9X02_11810	312	56285	57223	1		
236	*M. malmoense*	NZ_MBEC01000104.1	A5675_17775	302	8280	9188	1		
237	*M. malmoense*	NZ_MBED01000126.1	A5676_24675	302	115149	116057	1	7	7
238	*M. malmoense*	NZ_MBEE01000079.1	A5677_02250	302	183300	184208	1		
239	*M. malmoense*	NZ_MOWS01000083.1	BMG05_10105	295	38102	38989	1		
240	*M. malmoense*	NZ_MVHV01000020.1	BST29_18125	295	36578	37411	1		
241	*M. mantenii*	NZ_MVHW01000022.1	BST30_18185	301	21471	22376	1	1	1
242	*M. marinum*	NZ_ANPL01000002.1	MMEU_1727	291	246738	247613	2		
243	*M. marinum*	NC_010612.1	MMAR_4575	291	5614231	5615106	1	3	4
244	*M. marinum*	NZ_ANPM01000001.1	MMMB2_2935	291	3373280	3374155	1		
245	*M. marseillense*	NZ_MVHX01000013.1	BST31_11190	304	60104	61018	1	1	1
246	*M. microti*	CP010333.1	RN08_1045	273	1048507	1049328	1	1	1
247	*M. minnesotense*	NZ_MVHZ01000015.1	BST33_14090	288	103152	104018	1	1	1
248	*M. monacense*	NZ_MVIA01000011.1	BST34_11870	316	11602	12552	1	1	1
249	*M. moriokaense*	NZ_MVIB01000001.1	BST36_01090	307	225039	225962	1	1	1
250	*M. mucogenicum*	NZ_LSKL01000154.1	AX749_RS06970	279	27341	28180	2		
251	*M. mucogenicum*	NZ_LZLC01000055.1	A5630_15865	278	9475	10311	2	3	5
252	*M. mucogenicum*	NZ_CYSI01000007.1	BN2644_RS10735	277	2135842	2136675	1		
253	*M. mungi*	NZ_LXTB01000055.1	A7J32_07740	273	45089	45910	1	1	1
254	*M. nebraskense*	NZ_LQPH01000037.1	AWC17_25590	297	64067	64960	3	1	1

255	*M. neoaurum*	NZ_LQMX01000004.1	AVZ31_06500	309	121880	122809	3	2	5
256	*M. neoaurum*	NZ_JMDW01000003.1	IC40_RS0103340	309	313493	314422	2		
257	*M. nonchromogenicum*	NZ_LQPI01000087.1	AWC18_19855	292	7374	8252	1	1	1
258	*M. noviomagense*	NZ_MVIC01000010.1	BST37_08040	309	16448	17377	1	2	2
259	*M. novocastrense*	NZ_BCTA01000099.1	RMCN_5950	320	81866	82828	1		
260	*M. obuense*	NZ_LAUZ02000003.1	WN67_17740	317	17135	18088	1	2	2
261	*M. obuense*	NZ_JYNU01000020.1	MOBUDSM44075_03443	317	58259	59212	1		
262	*M. orygis*	NZ_APKD01000011.1	MORY_05401	273	84773	85594	1	1	1
263	*M. palustre*	NZ_LQPJ01000099.1	AWC19_08150	312	66036	66974	1	1	1
264	*M. paraense*	NZ_LQPK01000040.1	AWB91_26940	306	7958	8878	2	3	4
265	*M. paraense*	NZ_LQPM01000019.1	AWB89_19435	306	72026	72946	1		
266	*M. paraense*	NZ_LQPN01000072.1	AWB90_24295	306	209177	210097	1		
267	*M. paraffinicum*	NZ_MPNT01000001.1	BRW65_00370	302	86886	87794	1	1	1
268	*M. parafortuitum*	NZ_MVID01000016.1	BST38_17670	321	118025	118990	1	1	1
269	*M. paraintracellulare*	NZ_NCXN01000007.1	B8W68_07605	309	57293	58222	1	1	1
270	*M. parascrofulaceum*	NZ_GG770554.1	HMPREF0591_4750	312	334481	335419	1	1	1
271	*M. paraseoulense*	NZ_MVIE01000001.1	BST39_00680	297	152042	152935	1	1	1
272	*M. parmense*	NZ_LQPO01000035.1	AWC20_15670	302	158813	159721	1	1	1
273	*M. peregrinum*	NZ_LQPP01000042.1	AWC21_23415	297	21249	22142	2	4	5
274	*M. peregrinum*	NZ_LZIB01000029.1	A5719_16475	297	388622	389515	1		
275	*M. peregrinum*	NZ_LZSO01000016.1	A5792_16365	297	12966	13859	1		
276	*M. peregrinum*	NZ_LZSY01000187.1	A5779_10595	297	13456	14349	1		
277	*M. phlei*	NZ_ATHW01000044.1	MPHL43070_17445	300	42071	42973	6	1	6
278	*M. pinnipedii*	NZ_PYQH00000000.2	C9J59_005360	273	76212	77033	2	1	2
279	*M. porcinum*	NZ_MIHF01000134.1	BHQ19_19440	292	13690	14568	1	3	3
280	*M. porcinum*	NZ_MSTD01000001.1	BVU76_01470	291	277147	278022	1		
281	*M. porcinum*	NZ_MVIG01000003.1	BST41_07320	292	176038	176916	1		
282	*M. pseudoshottsii*	NZ_BCND01000075.1	MPS_4136	291	17004	17879	1	1	1
283	*M. rhodesiae*	NC_016604.1	MycrhN_3276	307	3275104	3276027	1	3	3
284	*M. rhodesiae*	NZ_AGIQ01000001.1	MycrhDRAFT_0595	298	579995	580891	1		
285	*M. rhodesiae*	NZ_MVIH01000004.1	BST42_10075	305	65992	66909	1		
286	*M. riyadhense*	NZ_LQPQ01000232.1	AWC22_05145	291	17376	18251	1	1	1
287	*M. rutilum*	NZ_LT629971.1	BLW81_RS12125	312	2468862	2469800	1	1	1
288	*M. salmoniphilum*	NZ_MAFE01000012.1	B4387_RS23720	269	54614	55423	1	2	2
289	*M. salmoniphilum*	NZ_MAFR01000055.1	B4385_RS22350	269	9811	10620	1		
290	*M. saopaulense*	NZ_MLIH01000036.1	BKG73_24695	269	188155	188964	2	2	3
291	*M. saopaulense*	NZ_MVII01000014.1	BST43_12430	269	67105	67914	1		
292	*M. saskatchewanense*	NZ_LQPR01000030.1	AWC23_14415	312	80235	81173	1	1	1
293	*M. scrofulaceum*	NZ_LZJW01000109.1	A5681_04615	302	28111	29019	1	3	3
294	*M. scrofulaceum*	NZ_LZJY01000154.1	A5679_13865	302	21319	22227	1		
295	*M. scrofulaceum*	NZ_MVIJ01000037.1	BST44_20875	302	14563	15471	1		
296	*M. senegalense*	NZ_LDCO01000015.1	AA982_17835	294	44226	45110	2	1	2
297	*M. senuense*	NZ_LQPS01000038.1	AWC24_13870	290	11743	12615	1	1	1

298	*M. septicum*	NZ_HG322952.1	TX88_RS23025	297	637324	638217	1	1	1
299	*M. setense*	NZ_JTJW01000010.1	QQ25_25720	297	93890	94783	1	2	2
300	*M. setense*	NZ_JTLZ01000010.1	QQ44_21925	297	96980	97873	1		
301	*M. sherrisii*	NZ_LQPT01000019.1	AWC25_21425	305	127905	128822	1	2	2
302	*M. sherrisii*	NZ_MIHC01000001.1	BHQ21_01225	305	238568	239485	1		
303	*M. shimoidei*	NZ_LQPU01000033.1	AWC26_14910	303	119734	120645	2	1	2
304	*M. shinjukuense*	NZ_MVIK01000079.1	BST45_19025	285	664	1521	1	1	1
305	*M. simiae*	NZ_CP010996.1	VC42_09630	317	2125891	2126844	1	3	3
306	*M. simiae*	NZ_HG315953.1	TY06_RS09920	311	2155416	2156351	1		
307	*M. simiae*	NZ_MZZM01000013.1	B5M45_07330	317	25093	26046	1		
308	*M. sinense*	NZ_LZKG01000093.1	A5710_22565	291	12584	13459	2	4	6
309	*M. sinense*	NC_015576.1	JDM601_RS04445	296	946711	947601	1		
310	*M. sinense*	NZ_LZIM01000080.1	A5772_11370	301	83993	84898	2		
311	*M. sinense*	NZ_LZMF01000126.1	A5648_09720	301	172446	173351	1		
312	*M. smegmatis*	NC_018289.1	MSMEI_5431	328	5670583	5671569	6	8	8
313	*M. smegmatis*	NZ_CM001762.1	D806_5651	327	5749283	5750266	1		
314	*M. smegmatis*	NZ_LN831039.1	ERS451418_05406	318	5674803	5675789	1		
315	*M. szulgai*	NZ_LQPW01000143.1	AWC27_07370	305	54390	55307	1	3	3
316	*M. szulgai*	NZ_LZHY01000027.1	A5725_16400	312	153422	154360	1		
317	*M. szulgai*	NZ_LZLW01000006.1	A5657_09495	312	960	1898	1		
318	*M. terrae*	NZ_LQPX01000045.1	AWC28_05745	296	315411	316301	1	1	1
319	*M. thermoresistibile*	NZ_BCTB01000009.1	RMCT_1700	309	302180	303109	2	1	2
320	*M. timonense*	NZ_MVIL01000001.1	BST46_RS00270	302	53580	54488	1	1	1
321	*M. triplex*	NZ_LQPY01000007.1	AWC29_06835	301	25389	26294	2	1	2
322	*M. triviale*	NZ_LQPZ01000017.1	AWC30_07580	275	49497	50324	1	2	2
323	*M. triviale*	NZ_MIHD01000004.1	BHQ15_04395	289	107866	108735	1		
324	*M. tuberculosis*	NC_000962.3	Rv0937c	273	1045199	1046020	5149		
325	*M. tuberculosis*	NZ_MQGD01000001.1	BOK91_RS05095	273	1047275	1048096	19		
326	*M. tuberculosis*	NZ_CM002074.1	M536_RS05175	273	1044848	1045669	9		
327	*M. tuberculosis*	NZ_FQBJ01000009.1	BT513_RS09790	273	18087	18908	6		
328	*M. tuberculosis*	NZ_CNEA01000008.1	AQ354_RS04675	273	18084	18905	5		
329	*M. tuberculosis*	NZ_KK356234.1	X108_00981	273	1052245	1053066	5		
330	*M. tuberculosis*	NZ_MMKQ01000002.1	BKS87_RS04920	273	704575	705396	5		
331	*M. tuberculosis*	NZ_KK331459.1	AO61_01234	273	137112	137933	4		
332	*M. tuberculosis*	NZ_KK337822.1	AP53_01250	273	123553	124374	4		
333	*M. tuberculosis*	NZ_CHIK01000012.1	AP747_RS07780	273	18084	18905	3		
334	*M. tuberculosis*	NZ_CNBV01000010.1	AYV50_RS03955	273	18083	18904	3		
335	*M. tuberculosis*	NZ_KK334477.1	AP04_RS06975	273	184632	185453	2		
336	*M. tuberculosis*	NZ_KK341749.1	V913_00977	273	1043931	1044752	2		
337	*M. tuberculosis*	NZ_MRGJ01000001.1	BS216_RS05095	273	1046259	1047080	2		
338	*M. tuberculosis*	NZ_AHHY01000763.1	MW9_RS20605	273	10816	11637	1		
339	*M. tuberculosis*	NZ_CHIV01000004.1	AQV47_RS04475	273	48133	48954	1		
340	*M. tuberculosis*	NZ_CHYH01000002.1	AP664_RS02795	273	204366	205187	1		

341	*M. tuberculosis*	NZ_CNBB01000010.1	AQ387_RS06380	273	76176	76997	1		
342	*M. tuberculosis*	NZ_CNBI01000012.1	AQ374_RS06865	273	18083	18904	1		
343	*M. tuberculosis*	NZ_CNBQ01000014.1	AQ273_RS07910	273	18083	18904	1	39	5243
344	*M. tuberculosis*	NZ_CNBR01000007.1	AQ247_RS04335	273	75408	76229	1		
345	*M. tuberculosis*	NZ_CNFM01000009.1	AQ320_RS05575	273	18084	18905	1		
346	*M. tuberculosis*	NZ_CNGA01000013.1	AQ151_RS07140	273	18083	18904	1		
347	*M. tuberculosis*	NZ_CNGF01000014.1	AQ142_RS07950	159	18087	18569	1		
348	*M. tuberculosis*	NZ_CNGO01000030.1	AQ079_RS10620	273	24091	24912	1		
349	*M. tuberculosis*	NZ_COQK01000010.1	AQ417_RS08730	273	51246	52067	1		
350	*M. tuberculosis*	NZ_COTU01000008.1	AQV06_RS06700	273	48185	49006	1		
351	*M. tuberculosis*	NZ_CP011510.1	TM57_RS10800	273	2295612	2296433	1		
352	*M. tuberculosis*	NZ_CQBD01000010.1	AD435_RS09635	273	49422	50243	1		
353	*M. tuberculosis*	NZ_CQSD01000006.1	AFA22_RS03640	273	80296	81117	1		
354	*M. tuberculosis*	NZ_FQAF01000012.1	BT291_RS12160	273	112958	113779	1		
355	*M. tuberculosis*	NZ_FUFS01000005.1	B1K90_RS02725	273	75532	76353	1		
356	*M. tuberculosis*	NZ_KK328322.1	K882_06632	273	2915251	2916072	1		
357	*M. tuberculosis*	NZ_KK336380.1	AP31_RS12620	273	137663	138484	1		
358	*M. tuberculosis*	NZ_KK339452.1	Q637_00981	273	1055427	1056248	1		
359	*M. tuberculosis*	NZ_KK353506.1	P923_02946	273	155420	156241	1		
360	*M. tuberculosis*	NZ_KK357796.1	X339_00690	273	713017	713838	1		
361	*M. tuberculosis*	NZ_KL405155.1	AL57_RS08550	273	113102	113923	1		
362	*M. tuberculosis*	NZ_MQII01000004.1	BOL62_RS05110	273	206178	206999	1		
363	*M. tusciae*	NZ_KI912270.1	MYCTUDRAFT_RS0205730	308	741371	742297	1	2	2
364	*M. tusciae*	NZ_MVIM01000021.1	BST47_26875	305	88400	89317	1		
365	*M. ulcerans*	NZ_MDUB01000121.1	A3649_20650	291	22969	23844	1	1	1
366	*M. vaccae*	NZ_BCRS01000191.1	MVA01S_RS26025	333	5151	6152	3	1	3
367	*M. vanbaalenii*	NC_008726.1	Mvan_4917	306	5268248	5269168	1	1	1
368	*M. vulneris*	NZ_MBEG01000085.1	A5722_29385	292	80795	81673	2		
369	*M. vulneris*	NZ_CCBG010000002.1	BN979_04906	292	1523810	1524409	1		
370	*M. vulneris*	NZ_MBDY01000095.1	A5717_31105	292	1506	2384	1	5	6
371	*M. vulneris*	NZ_MBEF01000046.1	A5721_11540	292	156295	157173	1		
372	*M. vulneris*	NZ_NCXM01000009.1	B8W69_10360	301	9964	10869	1		
373	*M. wolinskyi*	NZ_LGTW01000002.1	AFM11_03235	297	55609	56502	1	2	2
374	*M. wolinskyi*	NZ_LQQA01000010.1	AWC31_21850	297	420970	421863	1		
375	*M. xenopi*	NZ_AJFI01000095.1	MXEN_19119	309	207426	208355	1	2	2
376	*M. xenopi*	NZ_LQQB01000078.1	AWC32_03585	309	2501	3430	1		
377	*M. yongonense*	CP003347.1	OEM_09400	309	916154	917083	2		
378	*M. yongonense*	NZ_MBDX01000215.1	A5644_25960	309	15150	16079	1	3	4
379	*M. yongonense*	NZ_MBDZ01000039.1	A5689_11890	309	46595	47524	1		
