# Supplementary material for: A New Single Gene Differential Biomarker for Mycobacterium tuberculosis Complex and Non-tuberculosis Mycobacteria
Source: Front Microbiol. 2019 Aug 13;10:1887. doi: 10.3389/fmicb.2019.01887 (PMC6700215; doi:10.3389/fmicb.2019.01887)
Supplement: Supplementary file 2 [file Data_Sheet_2.PDF]

Supplementary table 2. Representative sequences of the *rpoB* genotypes in each Mycobacterium species/variants.

Numbers	Species/variants	Accession	Locus tag	Lehgth (bp)	Beginning of position	End of position	Strain number with this genotype	Total genotype number	Total strain number
1	*M. abscessus*	NZ_HE817967.1	MABCF_RS04860	3516	3925295	3928810	1		
2	*M. abscessus*	NZ_AJGF01000005.1	OUW_RS19160	3516	146923	150438	1		
3	*M. abscessus*	NZ_JMIA01000004.1	EG21_RS0117450	3516	447097	450612	1		
4	*M. abscessus*	NZ_JAOU01000001.1	NF24_RS14960	3516	1556749	1560264	1		
5	*M. abscessus*	NZ_CP014950.1	A3N95_RS18405	3516	3727136	3730651	1		
6	*M. abscessus*	NZ_CSWU01000002.1	AKZ87_RS06275	3516	445127	448642	1		
7	*M. abscessus*	NZ_CSZC01000002.1	AK122_RS07450	3514	445130	448643	1		
8	*M. abscessus*	NZ_CSZN01000001.1	AKZ39_RS02280	3515	453403	456917	1		
9	*M. abscessus*	NZ_CSZQ01000002.1	AK042_RS06600	3515	484918	488432	1		
10	*M. abscessus*	NZ_FSEW01000012.1	BUW78_RS20835	3516	41215	44730	1		
11	*M. abscessus*	NZ_FVQO01000002.1	B5508_RS04730	3516	199144	202659	1		
12	*M. abscessus*	NZ_FVBG01000005.1	B5408_RS14095	3516	296489	300004	1		
13	*M. abscessus*	NZ_FSGA01000005.1	BUR80_RS14260	3516	355320	351805	1		
14	*M. abscessus*	NZ_FVCH01000003.1	B5496_RS07180	3516	344590	348105	1		
15	*M. abscessus*	NZ_FSAT01000001.1	BUT64_RS02135	3516	434713	438228	1		
16	*M. abscessus*	NZ_FVWG01000001.1	B5Z01_RS05600	3516	1086924	1090439	1		
17	*M. abscessus*	NZ_FVVK01000001.1	B5046_RS05330	3516	1101294	1104809	1		
18	*M. abscessus*	NZ_FVXG01000001.1	B5278_RS02200	3516	449662	453177	1		
19	*M. abscessus*	NZ_FSMO01000001.1	BUT69_RS03205	3516	630018	633533	1		
20	*M. abscessus*	NZ_FSAN01000001.1	BUV59_RS08900	3516	1848484	1851999	1		
21	*M. abscessus*	NC_021282.1	MASS_RS19705	3516	3881534	3885049	1		
22	*M. abscessus*	NZ_FVNM01000003.1	B5203_RS12020	3516	400935	404450	1		
23	*M. abscessus*	NZ_FVFY01000001.1	B5149_RS03245	3516	639666	643181	1		
24	*M. abscessus*	NZ_FVTC01000002.1	B5062_RS04895	3516	148512	152027	1		
25	*M. abscessus*	NZ_FVPZ01000003.1	B5168_RS13380	3516	386050	389565	1		
26	*M. abscessus*	NZ_FVXU01000001.1	B5432_RS03405	3516	667801	671316	1		
27	*M. abscessus*	NZ_FVKE01000002.1	B5312_RS09440	3516	427442	430957	1		
28	*M. abscessus*	NZ_FVNF01000005.1	B5Y47_RS10245	3516	34748	38263	1		
29	*M. abscessus*	NZ_FVXD01000001.1	B5108_RS04740	3516	957267	960782	1		
30	*M. abscessus*	NZ_FVXW01000001.1	B5471_RS00535	3516	97331	100846	1		
31	*M. abscessus*	NZ_AUMY01000008.1	M879_RS01000000152345	3518	163166	166683	1		
32	*M. abscessus*	NZ_CP014961.1	A3O06_RS21470	3516	4229159	4232674	2		
33	*M. abscessus*	NZ_CSWN01000003.1	AKZ08_RS07235	3516	209572	213087	2		
34	*M. abscessus*	NZ_FSLE01000005.1	BUW44_RS13655	3516	154198	157713	2		
35	*M. abscessus*	NZ_FVNO01000001.1	B5Y21_RS07085	3516	1359518	1363033	2		
36	*M. abscessus*	NZ_FSLG01000001.1	BUW26_RS07005	3516	1346213	1349728	2		
37	*M. abscessus*	NZ_FSJJ01000001.1	BUT72_RS01890	3516	368033	371548	2		

38	*M. abscessus*	NZ_FVVE01000001.1	B5Y72_RS03345	3516	660844	664359	2		
39	*M. abscessus*	NZ_FSPL01000001.1	BUU54_RS03945	3516	810067	813582	2		
40	*M. abscessus*	NZ_FVFL01000001.1	B5328_RS02130	3516	437037	440552	2	80	1376
41	*M. abscessus*	NZ_FVCE01000001.1	B5161_RS04240	3516	797491	801006	2		
42	*M. abscessus*	NZ_FVMQ01000003.1	B5Z11_RS09400	3516	443092	446607	2		
43	*M. abscessus*	NZ_CSXF01000005.1	AKZ98_RS13655	3516	337186	340701	3		
44	*M. abscessus*	NZ_FVGD01000001.1	B5Y88_RS05220	3516	992120	995635	3		
45	*M. abscessus*	NZ_CP014960.1	A3O05_RS18565	3516	3770479	3773994	4		
46	*M. abscessus*	NZ_FSKL01000006.1	BUW65_RS17135	3516	316340	319855	4		
47	*M. abscessus*	NZ_AHAS01000016.1	MBOL_RS05570	3516	427213	430728	4		
48	*M. abscessus*	NZ_FSAX01000001.1	BUT54_RS07085	3516	1392024	1395539	4		
49	*M. abscessus*	NZ_FVKA01000001.1	B5Y39_RS04505	3516	928280	931795	4		
50	*M. abscessus*	NZ_FVCU01000003.1	B5116_RS14665	3516	475506	479021	4		
51	*M. abscessus*	NZ_FSBY01000001.1	BUW63_RS03620	3516	706417	709932	5		
52	*M. abscessus*	NZ_FSEA01000010.1	BUU15_RS16860	3516	132754	136269	5		
53	*M. abscessus*	NZ_FVLY01000001.1	B5390_RS10005	3516	1986626	1990141	5		
54	*M. abscessus*	NZ_FSDI01000003.1	BUS35_RS12800	3516	435678	439193	7		
55	*M. abscessus*	NZ_FSCN01000001.1	BUV47_RS04875	3516	947737	951252	7		
56	*M. abscessus*	NZ_FSKG01000003.1	BUW18_RS07195	3516	98376	101891	8		
57	*M. abscessus*	NZ_FVYW01000001.1	B5323_RS02145	3516	436820	440335	8		
58	*M. abscessus*	NZ_FVCC01000003.1	B5420_RS08485	3516	48023	51538	10		
59	*M. abscessus*	NZ_FVHN01000002.1	B5039_RS09410	3516	295061	298576	11		
60	*M. abscessus*	NZ_CP014952.1	A3N97_RS18650	3516	3782224	3785739	11		
61	*M. abscessus*	NZ_FVJY01000001.1	B5204_RS08595	3516	1698716	1702231	11		
62	*M. abscessus*	NZ_FSAR01000003.1	BUU30_RS12600	3516	147156	150671	11		
63	*M. abscessus*	NZ_CSUB01000001.1	AKY83_RS03910	3516	832709	836224	12		
64	*M. abscessus*	NZ_AKTX01000007.1	HJ82_RS10160	3516	1498679	1502194	15		
65	*M. abscessus*	NZ_AKVR01000001.1	Y1C_RS0103240	3516	635716	639231	15		
66	*M. abscessus*	NZ_CSTW01000004.1	AKY78_RS08370	3516	132105	135620	15		
67	*M. abscessus*	NZ_FSIM01000001.1	BUV80_RS03350	3516	662160	665675	15		
68	*M. abscessus*	NZ_FVWA01000003.1	B5Y65_RS13525	3516	495705	499220	15		
69	*M. abscessus*	NZ_AJMA01000002.1	UUO_RS0105545	3516	432503	436018	16		
70	*M. abscessus*	NZ_FWDD01000001.1	B6F62_RS05450	3516	1104374	1107889	19		
71	*M. abscessus*	NZ_CP016188.1	BAB74_RS18905	3516	3827796	3831311	25		
72	*M. abscessus*	NZ_FVOQ01000001.1	B5441_RS03385	3516	666685	670200	25		
73	*M. abscessus*	NZ_AJLZ01000001.1	UUM_RS0104055	3516	836500	840015	26		
74	*M. abscessus*	NZ_AJLY02000056.1	UUG_RS22710	3516	96847	100362	27		
75	*M. abscessus*	NZ_AJSD01000003.1	UUI_RS0102535	3516	5696	9211	28		
76	*M. abscessus*	NZ_CP014957.1	A3O02_RS19450	3516	3937931	3941446	38		
77	*M. abscessus*	NZ_KB290572.1	D498_RS0121425	3516	416626	420141	156		
78	*M. abscessus*	NZ_JRMD01000289.1	LJ55_RS18985	3516	17290	20805	191		
79	*M. abscessus*	NZ_AGQU01000001.1	MAB47J26_RS23360	3516	23251	26766	256		
80	*M. abscessus*	NZ_AKUX01000012.1	MA3A0119R_4171	3516	144916	148431	300		

81	*M. acapulcensis*	NZ_LT592225.1	BN8058_RS18355	3486	996918	1000403	1	1	1
82	*M. africanum*	NZ_KK338878.1	N057_RS09155	3519	758280	761798	1		
83	*M. africanum*	NZ_KK338951.1	N075_RS09190	3519	758476	761994	1		
84	*M. africanum*	NZ_KK339246.1	N109_RS09180	3519	758160	761678	1		
85	*M. africanum*	NZ_KK339269.1	N116_RS09175	3519	759905	763423	1		
86	*M. africanum*	NZ_KK338758.1	CH83_00695	3519	757727	761245	1	9	29
87	*M. africanum*	NZ_KK338784.1	CH86_RS09160	3519	758062	761580	1		
88	*M. africanum*	NZ_KK338837.1	N044_RS20030	3519	3169121	3172639	2		
89	*M. africanum*	NC_015758.1	MAF_RS03505	3519	757196	760714	3		
90	*M. africanum*	NZ_KK338483.1	BM98_RS09145	3519	758793	762311	18		
91	*M. algericum*	NZ_MVHC01000005.1	BST10_06860	3468	61671	65138	1	1	1
92	*M. alsense*	NZ_MVHD01000001.1	BST11_00260	3477	49464	52940	1	1	1
93	*M. angelicum*	NZ_MVHE01000010.1	BST12_09550	3516	57362	60877	1	1	1
94	*M. aromaticivorans*	NZ_JALN02000001.1	Y900_RS17410	3489	3634531	3638019	1	1	1
95	*M. arosiense*	NZ_MVHG01000006.1	BST14_04895	3435	123587	127021	1	1	1
96	*M. arupense*	NZ_MVHH01000051.1	BST15_RS17715	3555	2142	5696	2	1	2
97	*M. asiaticum*	NZ_LZLF01000058.1	A9W94_RS04565	3486	25778	29263	1		
98	*M. asiaticum*	NZ_LZKS01000154.1	A5661_RS22020	3486	47591	51076	1		
99	*M. asiaticum*	NZ_LZLR01000084.1	A5635_RS09220	3486	2679	6164	1		
100	*M. asiaticum*	NZ_LZMH01000069.1	A5645_RS19775	3486	217148	220633	1	7	9
101	*M. asiaticum*	NZ_LZLS01000002.1	A5634_RS00050	3486	6094	9579	1		
102	*M. asiaticum*	NZ_LZLQ01000096.1	A5636_RS15150	3486	108423	111908	1		
103	*M. asiaticum*	NZ_MVHI01000003.1	BST16_RS03240	3486	117492	120977	3		
104	*M. aurum*	NZ_LT549889.1	BN4366_RS28690	3495	6000028	6003522	1	2	2
105	*M. aurum*	NZ_CVQQ01000025.1	AURUM_RS25510	3501	75913	79413	1		
106	*M. austroafricanum*	NZ_HG964451.1	BN976_RS09685	3504	628270	631773	1	1	1
107	*M. avium*	NZ_LMVZ01000004.1	A4U34_RS03055	3435	86491	89925	1		
108	*M. avium*	NZ_AWNJ01000091.1	MAV3388_RS19810	3435	16059	19493	1		
109	*M. avium*	NZ_AYOB01000465.1	O973_RS51840	3435	4230	7664	1		
110	*M. avium*	NZ_ACFI01000197.1	MAAT_RS0120045	3435	15911	19345	1		
111	*M. avium*	NZ_LNBB01000001.1	A4U41_RS00345	3435	73370	76804	1		
112	*M. avium*	NZ_AYNY01000780.1	O972_RS52415	3434	4233	7666	1		
113	*M. avium*	NZ_BDOO01000002.1	B9C18_RS00865	3435	111869	115303	1		
114	*M. avium*	NZ_CP016396.1	BBJ32_RS06365	3435	1412924	1416358	1		
115	*M. avium*	NZ_BDNS01000027.1	B9B65_RS04670	3435	5603	9037	1		
116	*M. avium*	NZ_JAOP01000034.1	NF22_RS08755	3434	151505	154938	1		
117	*M. avium*	NZ_JAOD01000006.1	I548_RS07625	3444	432194	435637	1		
118	*M. avium*	NZ_AGAQ01000299.1	KEG_RS0112295	3468	1322	4876	1	23	145
119	*M. avium*	NZ_LNBL01000006.1	A4U12_RS00405	3435	13300	16734	2		
120	*M. avium*	NZ_BDNE01000134.1	B9B75_RS16035	3435	7026	10460	2		
121	*M. avium*	NZ_AYNT01000233.1	O982_RS50645	3435	8039	11473	2		
122	*M. avium*	NZ_BDNA01000022.1	B9B58_RS04790	3435	41154	44588	2		
123	*M. avium*	NZ_AFIF01000052.1	MAPs_39030	3435	16045	19479	3		

124	*M. avium*	NZ_BDNM01000061.1	B9A77_RS11445	3435	44663	48097	5		
125	*M. avium*	NZ_FKJL01000120.1	A8A86_RS17125	3435	101998	105432	14		
126	*M. avium*	NZ_BDNF01000100.1	B9A81_RS19980	3435	5633	9067	17		
127	*M. avium*	NZ_BDNB01000019.1	B9B72_RS04595	3435	136575	140009	24		
128	*M. avium*	NZ_AYNW01001140.1	O977_RS54295	3435	3383	6817	30		
129	*M. avium*	NZ_AYLW01000117.1	O984_RS49280	3435	8050	11484	32		
130	*M. bacteremicum*	NZ_MVHJ01000002.1	BST17_RS03305	3495	205922	209416	1	1	1
131	*M. boenickei*	NZ_LQOK01000013.1	AWB93_RS02700	3579	93605	97183	1	1	1
132	*M. bouchedurhonense*	NZ_MVHL01000001.1	BST19_00495	3435	89587	93021	1	1	1
133	*M. bovis*	NZ_AVSW01000033.1	O217_RS26650	3519	3968	7486	1		
134	*M. bovis*	NZ_KK308938.1	Z584_RS19380	3519	12761	16279	1		
135	*M. bovis*	NZ_JNAF01000056.1	EW34_RS38505	3519	152494	156012	1		
136	*M. bovis*	NZ_MINA01000093.1	BHM02_RS11200	3519	85056	88574	1	7	70
137	*M. bovis*	NZ_CDHE01000087.1	MBC5_RS16165	3518	152460	155977	1		
138	*M. bovis*	NZ_NBZZ01000007.1	B7S04_RS03615	3519	128537	132055	2		
139	*M. bovis*	NZ_AWPL01000015.1	O216_RS25925	3519	85018	88536	63		
140	*M. branderi*	NZ_MVHM01000001.1	BST20_04790	3507	916931	920437	1	1	1
141	*M. brisbanense*	NZ_BCSX01000024.1	APK37_RS14145	3510	335591	339100	1	1	1
142	*M. canariasense*	NZ_LQOL01000068.1	AWB94_24675	3495	72801	76295	2	1	2
143	*M. canettii*	NZ_CAON01000068.1	QZ52_RS03585	3519	81885	85403	1		
144	*M. canettii*	NC_015848.1	MCAN_RS03460	3519	769908	773426	1		
145	*M. canettii*	NC_019952.1	BN45_RS03615	3519	796254	799772	1	6	9
146	*M. canettii*	NC_019951.1	BN42_RS03545	3519	778557	782075	1		
147	*M. canettii*	NZ_CAOL01000080.1	QZ38_RS03625	3519	12061	15579	2		
148	*M. canettii*	NZ_CAOM01000056.1	QZ53_RS03530	3519	14460	17978	3		
149	*M. caprae*	NZ_CDHG01000011.1	MBO2_RS04705	3519	152360	155878	1	2	2
150	*M. caprae*	NZ_CP016401.1	BBG46_RS03735	3519	784235	787753	1		
151	*M. celatum*	NZ_BBUN01000134.1	AWU83_RS09090	3507	20979	24485	2	1	2
152	*M. celeriflavum*	NZ_MVHN01000005.1	BST21_RS07205	3486	208532	212017	1	1	1
153	*M. chelonae*	NZ_MLIL01000002.1	BKG77_RS20440	3489	3372361	3375849	1		
154	*M. chelonae*	NZ_MLIM01000023.1	BKG78_RS07165	3489	365111	368599	1		
155	*M. chelonae*	NZ_MLIN01000016.1	BKG79_RS17795	3489	45500	48988	1		
156	*M. chelonae*	NZ_MLIO01000012.1	BKG80_RS12680	3489	390544	394032	1		
157	*M. chelonae*	NZ_MLID01000011.1	BKG69_RS10420	3489	513639	517127	1		
158	*M. chelonae*	NZ_MAEU01000008.1	B4395_RS04695	3489	162645	166133	1		
159	*M. chelonae*	NZ_MLIJ01000016.1	BKG75_RS06390	3489	538928	542416	1		
160	*M. chelonae*	NZ_MAES01000006.1	B4392_RS08515	3489	230224	233712	1		
161	*M. chelonae*	NZ_MAEQ01000001.1	B4397_RS01590	3489	319423	322911	1	18	42
162	*M. chelonae*	NZ_MLIR01000002.1	BKG83_02235	3489	321328	324816	1		
163	*M. chelonae*	NZ_MLIS01000001.1	BKG84_RS16700	3489	3506627	3510115	1		
164	*M. chelonae*	NZ_MLIT01000014.1	BKG85_21110	3489	509842	513330	1		
165	*M. chelonae*	NZ_MAER01000013.1	B4381_RS08965	3489	170055	173543	2		
166	*M. chelonae*	NZ_MLIA01000011.1	BKG66_RS14530	3489	446315	449803	3		

167	*M. chelonae*	NZ_MLII01000056.1	BKG74_RS24995	3489	425975	429463	3		
168	*M. chelonae*	NZ_CP007220.1	BB28_RS19410	3489	3963786	3967274	4		
169	*M. chelonae*	NZ_MLCI01000002.1	BKG57_RS09430	3489	532822	536310	4		
170	*M. chelonae*	NZ_MLHZ01000012.1	BKG65_RS18680	3489	510276	513764	14		
171	*M. chimaera*	NZ_LT703505.1	CCZ12_RS24580	3534	5255193	5258726	9	1	1
172	*M. chlorophenolicum*	NZ_BCQY01000008.1	MCH01S_RS12370	3489	290290	293787	2	1	1
173	*M. chubuense*	NC_018027.1	MYCCH_RS04420	3498	912976	916473	1	2	3
174	*M. chubuense*	NZ_JYNX01000018.1	MCHUDSM44219_00858	3498	43591	47088	2		
175	*M. colombiense*	NZ_LT719128.1	B0530_RS02345	3447	446781	450227	1	15	17
176	*M. colombiense*	NZ_LZKI01000132.1	A5708_RS18220	3447	58593	62039	1		
177	*M. colombiense*	NZ_MBEP01000042.1	A5739_RS09835	3435	39893	43327	1		
178	*M. colombiense*	NZ_LZSX01000081.1	A5760_17990	3435	48235	51669	1		
179	*M. colombiense*	NZ_MBEI01000071.1	A5732_RS22830	3435	45997	49431	1		
180	*M. colombiense*	NZ_MBEN01000024.1	A5737_RS03635	3435	3945	7379	1		
181	*M. colombiense*	NZ_MBEO01000078.1	A5738_06870	3435	46651	50085	1		
182	*M. colombiense*	NZ_LZLH01000261.1	A5627_05125	3435	32229	35663	1		
183	*M. colombiense*	NZ_LZJS01000017.1	A5685_RS01755	3435	6392	9826	1		
184	*M. colombiense*	NZ_LZKZ01000192.1	A5621_RS12215	3435	15252	18686	1		
185	*M. colombiense*	NZ_LZMA01000256.1	A5653_RS26835	3435	62808	66242	1		
186	*M. colombiense*	NZ_LZLA01000399.1	A5620_RS23370	3435	6774	10208	1		
187	*M. colombiense*	NZ_LZKX01000280.1	A9W93_RS20010	3435	7991	11425	1		
188	*M. colombiense*	NZ_AFVW02000001.1	MCOL_V200075	3447	15993	19439	2		
189	*M. colombiense*	NZ_LZLG01000020.1	A5628_22350	3435	135127	138561	2		
190	*M. conceptionense*	NZ_MBGF01000120.1	A5743_RS25915	3513	98841	102353	1	5	11
191	*M. conceptionense*	NZ_LQOP01000034.1	AWB98_RS27365	3513	314463	317975	1		
192	*M. conceptionense*	NZ_LFOD01000056.1	ACT17_RS31530	3513	4100	7612	1		
193	*M. conceptionense*	NZ_LZLN01000259.1	A5639_RS30045	3513	4122	7634	3		
194	*M. conceptionense*	NZ_LZHX01000045.1	A5726_RS13875	3513	98707	102219	5		
195	*M. confluentis*	NZ_LQOQ01000001.1	AWB99_RS03850	3525	787298	790822	1	1	1
196	*M. conspicuum*	NZ_LQOR01000031.1	AWC00_RS13455	3435	64590	68024	1	2	2
197	*M. cosmeticum*	NZ_CCBB010000003.1	BN977_RS22180	3495	637069	640563	1		
198	*M. diernhoferi*	NZ_MPNS01000032.1	BRW64_RS28315	3480	10793	14272	2	1	2
199	*M. doricum*	NZ_LQOS01000049.1	AWC01_RS16575	3534	165644	169177	1	1	1
200	*M. elephantis*	NZ_MVHP01000002.1	BST23_RS02510	3486	95278	98763	1	2	2
201	*M. elephantis*	NZ_LBNO01000007.1	AAV95_RS04990	3486	94466	97951	1		
202	*M. engbaekii*	NZ_LQOT01000019.1	AWC02_RS05245	3549	86644	90192	1	1	1
203	*M. europaeum*	NZ_LQOU01000021.1	AWC03_RS11200	3450	91834	95283	1	1	1
204	*M. fallax*	NZ_LQOJ01000040.1	AWC04_RS11435	3525	57089	60613	1	1	1
205	*M. farcinogenes*	NZ_HG964481.1	BN975_RS10075	3513	2091303	2094815	1	1	1
206	*M. flavescens*	NZ_MIHA01000005.1	BHQ18_RS08560	3504	124859	128362	1	1	1
207	*M. florentinum*	NZ_LQOV01000015.1	AWC05_RS17620	3504	924819	928322	1	1	1
208	*M. fortuitum*	NZ_LZLO01000046.1	A5638_RS16290	3513	264771	268283	1		
209	*M. fortuitum*	NZ_LZKO01000008.1	A5664_RS01330	3513	124602	128114	1		

210	*M. fortuitum*	NZ_LZIW01000195.1	A5669_RS29730	3513	16562	20074	1		
211	*M. fortuitum*	NZ_BCSZ01000064.1	APK39_RS29155	3513	160535	164047	1		
212	*M. fortuitum*	NZ_CP011269.1	XA26_RS06755	3513	1429126	1432638	1		
213	*M. fortuitum*	NZ_LZSJ01000193.1	A5668_RS24730	3513	78305	81817	1		
214	*M. fortuitum*	NZ_LZLZ01000249.1	A5654_RS24005	3513	10018	13530	1		
215	*M. fortuitum*	NZ_LZSN01000079.1	A5763_RS16445	3513	11417	14929	1		
216	*M. fortuitum*	NZ_LZIP01000103.1	A5768_RS19870	3513	44054	47566	1	17	22
217	*M. fortuitum*	NZ_LZIV01000051.1	A5670_RS10925	3513	33006	36518	1		
218	*M. fortuitum*	NZ_LZLP01000083.1	A5637_RS18040	3525	178368	181892	1		
219	*M. fortuitum*	NZ_MBEK01000134.1	A5734_RS24225	3525	182749	186273	1		
220	*M. fortuitum*	NZ_JASW01000007.1	BI85_RS0104335	3516	303636	307151	1		
221	*M. fortuitum*	NZ_JH814728.1	MFORT_RS11225	3513	144958	148470	2		
222	*M. fortuitum*	NZ_LZKN01000043.1	A5666_RS08615	3513	45017	48529	2		
223	*M. fortuitum*	NZ_LZKM01000029.1	A5667_RS07735	3513	33245	36757	2		
224	*M. fortuitum*	NZ_LZIH01000018.1	A5751_RS00745	3513	74555	78067	3		
225	*M. fragae*	NZ_LQOW01000024.1	AWC06_RS11115	3525	46940	50464	1	1	1
226	*M. franklinii*	NZ_MLIK01000019.1	BKG76_RS15965	3489	1719302	1722790	1		
227	*M. franklinii*	NZ_MAFS01000002.1	B4388_RS02335	3504	75945	79448	1		
228	*M. franklinii*	NZ_MAFQ01000003.1	B4393_RS04790	3504	197542	201045	1	5	5
229	*M. franklinii*	NZ_MAEP01000003.1	B4399_RS10435	3504	503713	507216	1		
230	*M. franklinii*	NZ_MVHQ01000008.1	BST24_RS10520	3504	78902	82405	1		
231	*M. gastri*	NZ_LQOX01000010.1	AWC07_RS01980	3534	46380	49922	2	1	2
232	*M. genavense*	NZ_JAGZ01000017.1	T428_RS0123285	3435	15545	18979	1	1	1
233	*M. gilvum*	NC_009338.1	MFLV_RS25565	3525	5416895	5420419	1	2	2
234	*M. gilvum*	NC_014814.1	Mspyr1_45140	3525	4671775	4675299	1		
235	*M. goodii*	NZ_CP012150.1	AFA91_RS32015	3516	6842836	6846351	1	1	1
236	*M. gordonae*	NZ_MAEM01000566.1	A9W98_RS35275	3486	14088	17573	1		
237	*M. gordonae*	NZ_LKTM01000223.1	AO501_RS17310	3486	242235	245720	1	4	5
238	*M. gordonae*	NZ_LZLX01000308.1	A5656_RS27325	3486	6182	9667	1		
239	*M. gordonae*	NZ_MIHG01000175.1	BHQ23_RS25210	3486	6181	9666	2		
240	*M. haemophilum*	NZ_CP011883.2	B586_RS03455	3456	728627	732082	1	2	5
241	*M. haemophilum*	NZ_LDPT01000007.1	ABH36_RS07820	3456	36050	39505	4		
242	*M. hassiacum*	NZ_KB903885.1	A3G5_RS0119715	3504	71625	75128	2	1	1
243	*M. heckeshornense*	NZ_MPJF01000027.1	BMW24_RS10945	3525	6453	9977	2	2	3
244	*M. heidelbergense*	NZ_MVHR01000012.1	BST25_RS10665	3465	59230	62694	1		
245	*M. heraklionense*	NZ_LZME01000157.1	A5649_RS21480	3462	9477	12938	1		
246	*M. heraklionense*	NZ_LZLB01000051.1	A5631_RS09905	3549	17617	21165	1		
247	*M. heraklionense*	NZ_LZIU01000025.1	A5671_RS03505	3549	53116	56664	1	5	6
248	*M. heraklionense*	NZ_LDPO01000001.1	ABW16_RS03300	3549	662592	666140	1		
249	*M. heraklionense*	NZ_MBEL01000059.1	A5735_RS03745	3549	35958	39506	2		
250	*M. hiberniae*	NZ_LQOZ01000020.1	AWC09_RS06675	3462	37332	40793	1	1	1
251	*M. holsaticum*	NZ_MIGZ01000002.1	BHQ17_RS00770	3486	65188	68673	1	1	1
252	*M. houstonense*	NZ_FJVO01000099.1	BN4332_RS21170	3534	40488	44021	1	1	1

253	*M. icosiumassiliensis*	NZ_FJVP01000007.1	BN4346_RS01445	3549	164421	167969	1	1	1
254	*M. immunogenum*	NZ_LQYE01000001.1	AWB85_RS04220	3516	887066	890581	1	2	17
255	*M. immunogenum*	NZ_LQPA01000010.1	AWC10_RS09140	3516	6905	10420	16		
256	*M. indicus*	NC_018612.1	MIP_RS22040	3519	4809146	4812664	1	1	1
257	*M. insubricum*	NZ_MVHS01000036.1	BST26_RS14490	3483	35893	39375	1	1	1
258	*M. interjectum*	NZ_LQPB01000090.1	AWC11_RS25130	3444	138974	142417	1	2	2
259	*M. interjectum*	NZ_FJVQ01000009.1	BN4348_RS00685	3444	5126	8569	1		
260	*M. intermedium*	NZ_MVHT01000034.1	BST27_RS13920	3522	29863	33384	3	1	3
261	*M. intracellulare*	NZ_CP009499.1	LG41_RS20470	3447	4409301	4412747	1	10	13
262	*M. intracellulare*	NC_016948.1	OCQ_RS21975	3447	4729562	4733008	1		
263	*M. intracellulare*	NZ_LZIO01000134.1	A5769_RS19770	3447	42296	45742	1		
264	*M. intracellulare*	NZ_JAON01000043.1	RT25_RS24690	3462	461549	465010	1		
265	*M. intracellulare*	NZ_LZJT01000112.1	A5684_RS19890	3447	45326	48772	1		
266	*M. intracellulare*	NZ_LQPC01000020.1	AWC12_RS06215	3501	226602	230102	1		
267	*M. intracellulare*	NZ_AUWT01000007.1	N420_RS0103210	3501	44296	47796	1		
268	*M. intracellulare*	NZ_LWCS01000043.1	A4X20_RS22455	3501	208924	212424	1		
269	*M. intracellulare*	NZ_JAOM01000026.1	RT24_RS22175	3447	535470	538916	2		
270	*M. intracellulare*	NZ_ABIN01000293.1	MIA_RS22940	3447	16829	20275	3		
271	*M. iranicum*	NZ_LZJO01000082.1	A5690_RS09570	3462	16807	20268	1	1	1
272	*M. kansasii*	NZ_MVBN01000009.1	BZL29_RS25930	3446	43477	46922	1	11	21
273	*M. kansasii*	NZ_MVBM01000010.1	BZL30_RS27710	3446	21309	24754	1		
274	*M. kansasii*	NZ_CP019885.1	B1T47_RS09355	3447	2130796	2134242	1		
275	*M. kansasii*	NZ_JANY01000001.1	I547_RS05780	3374	1334357	1337730	1		
276	*M. kansasii*	NZ_LWCK01000083.1	A4G29_RS06785	3447	15841	19287	1		
277	*M. kansasii*	NZ_LWCH01000133.1	A4G26_RS11110	3447	42546	45992	1		
278	*M. kansasii*	NZ_LWCJ01000157.1	A4G27_RS26655	3555	16253	19807	1		
279	*M. kansasii*	NZ_LWCM01000085.1	A4G31_RS11260	3555	64953	68507	1		
280	*M. kansasii*	NZ_LWCI01000112.1	A4G28_RS12300	3555	46591	50145	1		
281	*M. kansasii*	NZ_JANZ01000001.1	I546_RS02155	3555	474107	477661	1		
282	*M. kansasii*	NZ_JAOA01000010.1	I545_RS23910	3447	207530	210976	11		
283	*M. koreense*	NZ_NCXO01000002.1	B8W67_RS01495	3519	104883	108401	1	1	1
284	*M. kubicae*	NZ_LQPD01000019.1	AWC13_RS03520	3522	78013	81534	1	1	1
285	*M. kumamotonense*	NZ_MVHU01000008.1	BST28_RS07805	3555	75274	78828	1	2	2
286	*M. kumamotonense*	NZ_LFOE01000012.1	ACT18_RS10570	3468	36549	40016	1		
287	*M. kyorinense*	NZ_LZKJ01000117.1	A5707_RS13080	3501	38222	41722	1	2	4
288	*M. kyorinense*	NZ_LQPE01000132.1	AWC14_RS16050	3507	91408	94914	3		
289	*M. lacus*	NZ_LQPF01000022.1	AWC15_RS03235	3537	77260	80796	1	1	1
290	*M. leprae*	NZ_LYPH01000031.1	A8144_RS05960	3537	23814	27350	4	1	4
291	*M. lepromatosis*	NZ_JRPY01000083.1	MLPM_RS09615	3444	68175	71618	1	1	1
292	*M. liflandii*	NC_020133.1	MULP_RS05255	3531	1211222	1214752	1	1	1
293	*M. litorale*	NZ_CP019882.1	B1R94_RS05045	3490	1043935	1047432	1	1	1
294	*M. llatzerense*	NZ_JXST01000003.1	TL10_RS02795	3504	88399	91902	1	2	2
295	*M. llatzerense*	NZ_LIPZ01000004.1	BMI94_RS02665	3504	31727	35230	1		

296	*M. longobardum*	NZ_LQPG01000033.1	AWC16_RS17010	3459	21734	25192	1	1	1
297	*M. mageritense*	NZ_CCBF010000001.1	BN978_RS04915	3513	1011042	1014554	1	2	2
298	*M. mageritense*	NZ_AGSZ01000462.1	MFOR_RS21225	3513	4040	7552	1		
299	*M. malmoense*	NZ_MOWS01000054.1	BMG05_RS04395	3474	23179	26652	1	6	7
300	*M. malmoense*	NZ_MBEE01000136.1	A5677_RS14290	3540	22347	25886	1		
301	*M. malmoense*	NZ_MBEA01000168.1	A5674_RS14440	3450	9282	12731	1		
302	*M. malmoense*	NZ_MBEB01000363.1	A9X02_RS15725	3450	7470	10919	1		
303	*M. malmoense*	NZ_MVHV01000002.1	BST29_RS02550	3474	171024	174497	1		
304	*M. malmoense*	NZ_MBED01000020.1	A5676_RS02615	3450	9282	12731	2		
305	*M. mantenii*	NZ_MVHW01000003.1	BST30_RS03670	3450	112591	116040	1	1	1
306	*M. marinum*	NZ_HG917972.2	MMARE11_RS04700	3531	1157926	1161456	1	4	4
307	*M. marinum*	NC_010612.1	MMAR_RS04900	3531	1213006	1216536	1		
308	*M. marinum*	NZ_ANPM01000002.1	MMMB2_RS21455	3531	645868	649398	1		
309	*M. marinum*	NZ_ANPL01000004.1	MMEU_RS23345	3531	169923	173453	1		
310	*M. marseillense*	NZ_MVHX01000018.1	BST31_RS13950	3447	58985	62431	1	1	1
311	*M. minnesotense*	NZ_MVHZ01000002.1	BST33_RS03450	3555	361674	365228	1	1	1
312	*M. monacense*	NZ_MVIA01000030.1	BST34_RS21015	3519	3143	6661	1	1	1
313	*M. moriokaense*	NZ_MVIB01000024.1	BST36_RS22165	3486	100463	103948	1	1	1
314	*M. mucogenicum*	NZ_LZLC01000039.1	A5630_RS04395	3504	64191	67694	1	4	5
315	*M. mucogenicum*	NZ_LZSF01000006.1	A5642_RS00330	3504	22705	26208	1		
316	*M. mucogenicum*	NZ_CYSI01000007.1	BN2644_RS21265	3504	4324826	4328329	1		
317	*M. mucogenicum*	NZ_LSKA01000256.1	AX746_RS15140	3504	6748	10251	2		
318	*M. mungi*	NZ_LXTB01000087.1	A7J32_RS18245	3519	35121	38639	1	1	1
319	*M. nebraskense*	NZ_LDPP01000030.1	ABW17_RS19615	3435	46270	49704	1	2	3
320	*M. nebraskense*	NZ_LASX01000150.1	WU83_RS17055	3372	47	3418	2		
321	*M. neoaurum*	NZ_JMDW01000008.1	IC40_RS0109140	3495	342542	346036	2	2	5
322	*M. neoaurum*	NZ_LQMX01000001.1	AVZ31_RS00810	3480	162024	165503	3		
323	*M. nonchromogenicum*	NZ_LQPI01000033.1	AWC18_RS07040	3549	143884	147432	1	1	1
324	*M. noviomagense*	NZ_MVIC01000024.1	BST37_RS13715	3525	57457	60981	1	2	2
325	*M. novocastrense*	NZ_BCTA01000056.1	APK40_RS20595	3486	43222	46707	1		
326	*M. obuense*	NZ_JYNU01000057.1	MOBUDSM44075_04537	3495	279974	283468	1	2	2
327	*M. obuense*	NZ_LAUZ02000004.1	WN67_RS03060	3495	97910	101404	1		
328	*M. orygis*	NZ_APKD01000007.1	MORY_RS25785	3525	152397	155921	1	1	1
329	*M. palustre*	NZ_LQPJ01000147.1	AWC19_RS26700	3516	42757	46272	1	1	1
330	*M. paraense*	NZ_LQPM01000030.1	AWB89_RS21500	3456	96084	99539	1	2	2
331	*M. paraense*	NZ_LQPN01000053.1	AWB90_RS16665	3456	96459	99914	1		
332	*M. paraense*	NZ_LQPK01000022.1	AWB91_RS20685	3456	140651	144106	2	1	2
333	*M. paraffinicum*	NZ_MPNT01000025.1	BRW65_RS22155	3525	9900	13424	1	1	1
334	*M. parafortuitum*	NZ_MVID01000028.1	BST38_RS23790	3504	35066	38569	1	1	1
335	*M. paraintracellulare*	NZ_NCXN01000038.1	B8W68_RS21820	3447	800	4246	1	1	1
336	*M. parascrofulaceum*	NZ_GG770553.1	HMPREF0591_RS07190	3522	1520762	1524283	1	1	1
337	*M. paraseoulense*	NZ_MVIE01000010.1	BST39_RS10235	3450	49291	52740	1	1	1
338	*M. parmense*	NZ_LQPO01000084.1	AWC20_RS26550	3534	320806	324339	1	1	1

339	*M. peregrinum*	NZ_LZSY01000158.1	A5779_RS24455	3510	31010	34519	1	5	5
340	*M. peregrinum*	NZ_LZSO01000028.1	A5792_RS17500	3510	195402	198911	1		
341	*M. peregrinum*	NZ_LZIB01000035.1	A5719_RS16675	3510	122774	126283	1		
342	*M. peregrinum*	NZ_LN879427.1	BN2643_RS02375	3510	371662	375171	1		
343	*M. peregrinum*	NZ_LQPP01000030.1	AWC21_RS16275	3510	112893	116402	1		
344	*M. phlei*	NZ_ATHW01000041.1	MPHL43070_RS12440	3483	143380	146862	5	1	5
345	*M. porcinumce*	NZ_MIHF01000079.1	BHQ19_RS13920	3510	19222	22731	1	2	2
346	*M. porcinumce*	NZ_MSTD01000016.1	BVU76_RS13775	3510	83565	87074	1		
347	*M. porcinumce*	NZ_MVIG01000001.1	BST41_RS00405	3510	84092	87601	1	1	1
348	*M. pseudoshottsii*	NZ_BCND01000021.1	MPS_RS08540	3531	61848	65378	1	1	1
349	*M. rhodesiae*	NZ_AGIQ01000002.1	MYCRHDRAFT_RS17300	3498	1661753	1665250	1	1	1
350	*M. rhodesiae*	NC_016604.1	MYCRHN_RS03655	3513	760970	764482	1	2	2
351	*M. rhodesiae*	NZ_MVIH01000008.1	BST42_RS17295	3489	32941	36429	1		
352	*M. riyadhense*	NZ_LQPQ01000029.1	AWC22_RS03850	3534	74648	78181	1	1	1
353	*M. rutilum*	NZ_LT629971.1	BLW81_RS00250	3486	51944	55429	1	1	1
354	*M. salmoniphilum*	NZ_MAFE01000001.1	B4387_RS02230	3516	457393	460908	1	2	2
355	*M. salmoniphilum*	NZ_MAFR01000002.1	B4385_RS01145	3489	41653	45141	1		
356	*M. saopaulense*	NZ_MLIC01000001.1	BKG68_RS02120	3516	441398	444913	3	1	3
357	*M. saskatchewanense*	NZ_LQPR01000076.1	AWC23_RS24165	3435	42362	45796	1	1	1
358	*M. scrofulaceum*	NZ_MVIJ01000008.1	BST44_RS07715	3549	106597	110145	1	2	2
359	*M. scrofulaceum*	NZ_LZJY01000485.1	A5679_RS27575	3525	8384	11908	1		
360	*M. scrofulaceum*	NZ_LZJW01000087.1	A5681_RS13170	3540	45846	49385	1	1	1
361	*M. senegalense*	NZ_LDCO01000067.1	AA982_RS32290	3513	518	4030	2	1	2
362	*M. senuense*	NZ_LQPS01000051.1	AWC24_RS19335	3549	8209	11757	1	1	1
363	*M. septicum*	NZ_HG322951.1	TX88_RS08470	3510	1806406	1809915	1	1	1
364	*M. setense*	NZ_LZSL01000014.1	A5761_RS04855	3510	56302	59811	1	3	3
365	*M. setense*	NZ_JTJW01000005.1	QQ25_RS07445	3510	251938	255447	1		
366	*M. setense*	NZ_JTLZ01000004.1	QQ44_RS04900	3510	107329	110838	1		
367	*M. sherrisii*	NZ_LQPT01000069.1	AWC25_RS10730	3435	16105	19539	1	2	2
368	*M. sherrisii*	NZ_MIHC01000009.1	BHQ21_RS06895	3435	16090	19524	1		
369	*M. shimoidei*	NZ_LQPU01000062.1	AWC26_RS22015	3534	108830	112372	2	1	2
370	*M. shinjukuense*	NZ_MVIK01000002.1	BST45_RS01605	3537	147100	150636	1	1	1
371	*M. simiae*	NZ_HG315953.1	TY06_RS19115	3504	4042516	4046019	1	3	3
372	*M. simiae*	NZ_CP010996.1	VC42_RS18670	3504	4004814	4008317	1		
373	*M. simiae*	NZ_MZZM01000016.1	B5M45_RS11515	3504	234036	237539	1		
374	*M. sinense*	NZ_LZMF01000102.1	A5648_RS15080	3468	28493	31960	1	4	6
375	*M. sinense*	NC_015576.1	JDM601_RS03390	3555	722532	726086	1		
376	*M. sinense*	NZ_LZIN01000066.1	A5771_RS12840	3555	28039	31593	2		
377	*M. sinense*	NZ_LZJK01000018.1	A5694_RS01135	3468	19383	22850	2		
378	*M. smegmatis*	NZ_LN831039.1	AT701_RS07130	3510	1538799	1542308	1	3	8
379	*M. smegmatis*	NZ_CM001762.1	D806_RS06975	3510	1513854	1517363	1		
380	*M. smegmatis*	NZ_KI929247.1	AD56_RS0127920	3510	57678	61187	6		
381	*M. szulgai*	NZ_LZLW01000005.1	A5657_RS00480	3522	51129	54650	1		

382	*M. szulgai*	NZ_LZHY01000051.1	A5725_RS23875	3522	244610	248131	1	3	3
383	*M. szulgai*	NZ_LQPW01000031.1	AWC27_RS08195	3516	234272	237787	1		
384	*M. terrae*	NZ_LQPX01000002.1	AWC28_RS00340	3474	11969	15442	1	1	1
385	*M. thermoresistibile*	NZ_AGVE01000046.1	KEK_RS12400	3507	499552	503058	2	1	2
386	*M. timonense*	NZ_MVIL01000032.1	BST46_RS11855	3435	6553	9987	1	1	1
387	*M. triplex*	NZ_LQPY01000033.1	AWC29_RS20910	3435	50921	54355	2	1	2
388	*M. triviale*	NZ_LQPZ01000021.1	AWC30_RS09285	3510	41065	44574	1	2	2
389	*M. triviale*	NZ_MIHD01000016.1	BR336_RS11955	3519	67695	71213	1		
390	*M. tuberculosis*	NZ_MMZZ01000002.1	BKR43_RS03470	3519	649679	653197	1		
391	*M. tuberculosis*	NZ_MMIM01000003.1	BKT43_RS03495	3519	422989	426507	1		
392	*M. tuberculosis*	NZ_COGH01000002.1	AQU21_RS02410	3519	152445	155963	1		
393	*M. tuberculosis*	NZ_CFTQ01000003.1	AYW00_RS02540	3519	74012	77530	1		
394	*M. tuberculosis*	NZ_COUE01000001.1	AQ411_RS00420	3519	74099	77617	1		
395	*M. tuberculosis*	NZ_COUT01000001.1	AQV59_RS00425	3519	74085	77603	1		
396	*M. tuberculosis*	NZ_COLF01000002.1	AQV45_RS01915	3519	125774	129292	1		
397	*M. tuberculosis*	NZ_CPAE01000001.1	AQY12_RS00695	3519	142304	145822	1		
398	*M. tuberculosis*	NZ_CPIY01000003.1	AQY90_RS03045	3519	73958	77476	1		
399	*M. tuberculosis*	NZ_CNDU01000011.1	AQ328_RS08240	3519	17818	21336	1		
400	*M. tuberculosis*	NZ_CNED01000013.1	AQ210_RS07725	3519	69999	73517	1		
401	*M. tuberculosis*	NZ_CNEH01000008.1	AYV64_RS04675	3519	24113	27631	1		
402	*M. tuberculosis*	NZ_CNEO01000010.1	AQ315_RS05740	3519	12049	15567	1		
403	*M. tuberculosis*	NZ_CNER01000010.1	AQ281_RS05415	3519	14165	17683	1		
404	*M. tuberculosis*	NZ_CNEW01000010.1	AQ097_RS05690	3519	70202	73720	1		
405	*M. tuberculosis*	NZ_CFJH01000012.1	AP791_RS06545	3518	70035	73552	1		
406	*M. tuberculosis*	NZ_CNGB01000008.1	ERS027648_01143	3519	72885	76403	1		
407	*M. tuberculosis*	NZ_CNII01000011.1	AQZ65_RS07285	3519	70136	73654	1		
408	*M. tuberculosis*	NZ_CIKK01000011.1	AYV60_RS06450	3519	24138	27656	1		
409	*M. tuberculosis*	NZ_CNLR01000012.1	AQ124_RS06580	3519	70205	73723	1		
410	*M. tuberculosis*	NZ_CNBR01000009.1	AQ247_RS05260	3519	70140	73658	1		
411	*M. tuberculosis*	NZ_CNAX01000011.1	AQ221_RS06015	3519	12031	15549	1		
412	*M. tuberculosis*	NZ_MMCY01000002.1	BKW98_RS03480	3519	419773	423291	1		
413	*M. tuberculosis*	NZ_MMBR01000001.1	BKX32_RS03490	3519	763517	767035	1		
414	*M. tuberculosis*	NZ_CGCU01000009.1	AP320_RS04795	3519	70090	73608	1		
415	*M. tuberculosis*	NZ_MMMW01000001.1	BKU43_RS03495	3519	760183	763701	1		
416	*M. tuberculosis*	NZ_CQTS01000009.1	AKV07_RS04215	3519	17736	21254	1		
417	*M. tuberculosis*	NZ_CQUD01000027.1	AKS40_RS09610	3519	12062	15580	1		
418	*M. tuberculosis*	NZ_KK328401.1	K904_02946	3519	3161810	3165328	1		
419	*M. tuberculosis*	NZ_KK340419.1	T626_02916	3519	3116487	3120005	1		
420	*M. tuberculosis*	NZ_KK307322.1	Z562_00621	3519	40567	44085	1		
421	*M. tuberculosis*	NZ_JKBV01000002.1	Z547_00353	3519	153288	156806	1		
422	*M. tuberculosis*	NZ_JKBC01000001.1	Z567_00197	3519	187602	191120	1		
423	*M. tuberculosis*	NZ_KK306680.1	Z552_RS08120	3519	153299	156817	1		
424	*M. tuberculosis*	NZ_JKBR01000008.1	Z551_RS05010	3519	41679	45197	1		

425	*M. tuberculosis*	NZ_KK323942.1	Z550_00995	3519	34891	38409	1
426	*M. tuberculosis*	NZ_KK308166.1	Z576_01372	3519	86256	89774	1
427	*M. tuberculosis*	NZ_KK356123.1	X100_00696	3519	762272	765790	1
428	*M. tuberculosis*	NZ_KK338574.1	BO02_02982	3519	762559	766077	1
429	*M. tuberculosis*	NZ_FWED01000026.1	CK97_RS05515	3536	72707	76242	1
430	*M. tuberculosis*	NZ_KK353798.1	P966_02225	3519	2358818	2362336	1
431	*M. tuberculosis*	NZ_KK353912.1	P978_02479	3519	758829	762347	1
432	*M. tuberculosis*	NZ_KK330417.1	AO46_RS07750	3519	136931	140449	1
433	*M. tuberculosis*	NZ_KK331872.1	AO67_RS09775	3519	153926	157444	1
434	*M. tuberculosis*	NZ_KK338995.1	N079_RS18795	3519	3013	6531	1
435	*M. tuberculosis*	NZ_KK339284.1	N119_00696	3519	763456	766974	1
436	*M. tuberculosis*	NZ_KL406342.1	N129_02170	3519	153384	156902	1
437	*M. tuberculosis*	NZ_KK312744.1	AJ11_00733	3519	17770	21288	1
438	*M. tuberculosis*	NZ_DF126614.1	NCGM2209_RS06200	3519	1238408	1241926	1
439	*M. tuberculosis*	NZ_KK327868.1	I106_RS19875	3519	3174480	3177998	1
440	*M. tuberculosis*	NZ_CM002052.1	M389_RS03635	3519	759875	763393	1
441	*M. tuberculosis*	NZ_MMGG01000001.1	BKP20_RS03475	3519	756841	760359	1
442	*M. tuberculosis*	NZ_MREE01000002.1	BS158_RS03590	3519	607442	610960	1
443	*M. tuberculosis*	NZ_MMHP01000001.1	BKO85_RS03495	3519	764566	768084	1
444	*M. tuberculosis*	NZ_MKFW01000001.1	BI363_RS03470	3519	757252	760770	1
445	*M. tuberculosis*	NZ_MMGZ01000002.1	BKP01_RS03490	3519	422835	426353	1
446	*M. tuberculosis*	NZ_MMKS01000005.1	BKS85_RS03515	3519	86277	89795	1
447	*M. tuberculosis*	NZ_MLFI01000001.1	BJF75_RS03605	3519	761654	765172	1
448	*M. tuberculosis*	NZ_MMGD01000001.1	BKP23_RS03495	3519	761893	765411	1
449	*M. tuberculosis*	NZ_MMKN01000004.1	BKS90_RS03495	3519	421697	425215	1
450	*M. tuberculosis*	NZ_MMPZ01000004.1	BKV73_RS03490"	3519	421719	425237	1
451	*M. tuberculosis*	NZ_MMGQ01000001.1	BKP10_RS03475	3519	760541	764059	1
452	*M. tuberculosis*	NZ_MMSZ01000002.1	BKU94_RS03495	3519	422940	426458	1
453	*M. tuberculosis*	NZ_MMWB01000001.1	BKS45_RS03480	3519	756080	759598	1
454	*M. tuberculosis*	NZ_COHB01000007.1	AQT18_RS06385	3519	152453	155971	1
455	*M. tuberculosis*	NZ_MMQM01000008.1	BKV59_RS03525	3519	86938	90456	1
456	*M. tuberculosis*	NZ_COLU01000003.1	AQX45_RS02490	3519	73170	76688	1
457	*M. tuberculosis*	NZ_CFTI01000002.1	AP511_RS01660	3519	73940	77458	1
458	*M. tuberculosis*	NZ_CPER01000004.1	AQ051_RS03990	3519	126709	130227	1
459	*M. tuberculosis*	NZ_CPEH01000002.1	AQY70_RS01820	3519	74013	77531	1
460	*M. tuberculosis*	NZ_FQBY01000012.1	BT393_RS10420	3519	17777	21295	1
461	*M. tuberculosis*	NZ_FQOW01000009.1	BT881_RS09000	3519	125965	129483	1
462	*M. tuberculosis*	NZ_FQOZ01000006.1	BT908_RS06840	3519	24112	27630	1
463	*M. tuberculosis*	NZ_FPVC01000014.1	BT717_RS11665	3519	17769	21287	1
464	*M. tuberculosis*	NZ_FPWE01000016.1	BT699_RS14430	3519	17237	20755	1
465	*M. tuberculosis*	NZ_FPQP01000016.1	BT737_RS13780	3519	87395	90913	1
466	*M. tuberculosis*	NZ_FPWP01000001.1	BT420_RS00815	3519	143637	147155	1
467	*M. tuberculosis*	NZ_FQOE01000008.1	BT962_RS07710	3519	17778	21296	1

468	*M. tuberculosis*	NZ_MMUV01000004.1	BKS77_RS03495	3519	86805	90323	1
469	*M. tuberculosis*	NZ_MREH01000001.1	BS161_RS03595	3519	761692	765210	1
470	*M. tuberculosis*	NZ_CQFA01000004.1	AEF53_RS00810	3519	29816	33334	1
471	*M. tuberculosis*	NZ_CQFO01000010.1	AD282_RS05090	3519	70055	73573	1
472	*M. tuberculosis*	NZ_CQFR01000015.1	AEN20_RS07875	3519	13170	16688	1
473	*M. tuberculosis*	NZ_CNDL01000015.1	AQ338_RS09325	3519	70024	73542	1
474	*M. tuberculosis*	NZ_CNEJ01000008.1	AQ159_RS05545	3519	73779	77297	1
475	*M. tuberculosis*	NZ_CHWO01000010.1	AP706_RS06850	3519	70124	73642	1
476	*M. tuberculosis*	NZ_CNCR01000008.1	AQ275_RS03955	3519	69935	73453	1
477	*M. tuberculosis*	NZ_CNHX01000012.1	AQ175_RS06625	3519	70018	73536	1
478	*M. tuberculosis*	NZ_CNIJ01000005.1	AQ041_RS04260	3519	70049	73567	1
479	*M. tuberculosis*	NZ_CNIL01000009.1	AQ006_RS05515	3519	73496	77014	1
480	*M. tuberculosis*	NZ_CNIT01000011.1	AQ014_RS06095	3519	70049	73567	1
481	*M. tuberculosis*	NZ_CNIW01000011.1	AQ235_RS06620	3519	70028	73546	1
482	*M. tuberculosis*	NZ_CNLV01000013.1	AQ169_RS07380	3519	70064	73582	1
483	*M. tuberculosis*	NZ_CNDD01000012.1	AQ277_RS06275	3519	67571	71089	1
484	*M. tuberculosis*	NZ_CNMV01000012.1	AQ029_RS07915	3519	73753	77271	1
485	*M. tuberculosis*	NZ_CNCG01000010.1	AQ208_RS04870	3519	70052	73570	1
486	*M. tuberculosis*	NZ_CNCI01000007.1	AQ284_RS05075	3519	24083	27601	1
487	*M. tuberculosis*	NZ_CNCL01000015.1	AQ377_RS08105	3519	69930	73448	1
488	*M. tuberculosis*	NZ_CFSB01000014.1	AP848_RS08020	3519	69988	73506	1
489	*M. tuberculosis*	NZ_JHUF01000008.1	MTG12_RS0103880	3519	4094	7612	1
490	*M. tuberculosis*	NZ_MQKR01000001.1	BOK50_RS03590	3519	758763	762281	1
491	*M. tuberculosis*	NZ_MQCC01000001.1	BOJ57_RS03595	3519	758614	762132	1
492	*M. tuberculosis*	NZ_MQHV01000003.1	BOL49_RS03615	3519	87829	91347	1
493	*M. tuberculosis*	NZ_MQIJ01000007.1	BOL63_RS03630	3519	86677	90195	1
494	*M. tuberculosis*	NZ_MSLU01000041.1	BUN19_RS09035	3519	85360	88878	1
495	*M. tuberculosis*	NZ_JUFF01000009.1	PO07_RS03925	3519	17727	21245	1
496	*M. tuberculosis*	NZ_CQSL01000043.1	AKR80_RS13310	3519	23223	26741	1
497	*M. tuberculosis*	NZ_KK355346.1	W077_RS07700	3519	427929	431447	1
498	*M. tuberculosis*	NZ_KK316941.1	AL64_00696	3519	758343	761861	1
499	*M. tuberculosis*	NZ_KK320897.1	AM87_RS07840	3519	152672	156190	1
500	*M. tuberculosis*	NZ_KK324619.1	AN23_RS08220	3519	153906	157424	1
501	*M. tuberculosis*	NZ_KK328070.1	K853_00808	3519	868080	871598	1
502	*M. tuberculosis*	NZ_KK328260.1	K872_RS14925	3519	1229394	1232912	1
503	*M. tuberculosis*	NZ_KK327763.1	G065_04882	3519	1226215	1229733	1
504	*M. tuberculosis*	NZ_KK339662.1	T579_RS16230	3519	2355379	2358897	1
505	*M. tuberculosis*	NZ_KK322726.1	X433_RS07595	3519	422420	425938	1
506	*M. tuberculosis*	NZ_APHO01000007.1	G417_RS0100850	3519	3162	6680	1
507	*M. tuberculosis*	NZ_KK306763.1	Z555_RS05915	3519	39376	42894	1
508	*M. tuberculosis*	NZ_CP002882.1	HKBT2_RS03490	3519	758814	762332	1
509	*M. tuberculosis*	NZ_KK355985.1	X096_RS15950	3519	153953	157471	1
510	*M. tuberculosis*	NZ_KK356485.1	X125_RS11045	3519	1240788	1244306	1

511	*M. tuberculosis*	NZ_KK356641.1	X154_RS07390	3519	329421	332939	1
512	*M. tuberculosis*	NZ_KK356970.1	X182_RS09220	3519	759864	763382	1
513	*M. tuberculosis*	NZ_KK357251.1	X201_RS09180	3519	765181	768699	1
514	*M. tuberculosis*	NZ_KL405913.1	AP56_RS06860	3519	153817	157335	1
515	*M. tuberculosis*	NZ_KL406012.1	AP52_RS12720	3519	105862	109380	1
516	*M. tuberculosis*	NZ_KK340533.1	V196_RS12710	3519	150258	153776	1
517	*M. tuberculosis*	NZ_KL407303.1	P927_00696	3519	762511	766029	1
518	*M. tuberculosis*	NZ_KK353555.1	P929_RS09090	3519	757046	760564	1
519	*M. tuberculosis*	NZ_KK353559.1	P930_RS07810	3519	424184	427702	1
520	*M. tuberculosis*	NZ_KK353726.1	P954_RS15040	3519	772400	775918	1
521	*M. tuberculosis*	NZ_KK353876.1	P975_01720	3519	1861910	1865428	1
522	*M. tuberculosis*	NZ_KK353925.1	P979_00697	3519	757854	761372	1
523	*M. tuberculosis*	NZ_KL407292.1	P980_01587	3519	421218	424736	1
524	*M. tuberculosis*	NZ_KK321637.1	AN46_RS11345	3519	85492	89010	1
525	*M. tuberculosis*	NZ_KK321927.1	AN60_RS09230	3519	153481	156999	1
526	*M. tuberculosis*	NZ_KK327307.1	BA40_RS11450	3519	85121	88639	1
527	*M. tuberculosis*	NZ_KK327536.1	BA61_RS16220	3519	56272	59790	1
528	*M. tuberculosis*	NZ_MMOJ01000001.1	BKW15_RS03475	3519	756423	759941	1
529	*M. tuberculosis*	NZ_MMLM01000003.1	BKT89_RS03495	3519	422543	426061	1
530	*M. tuberculosis*	NZ_MREO01000005.1	BS169_RS03610	3519	421725	425243	1
531	*M. tuberculosis*	NZ_MMZY01000001.1	BKR44_RS03465	3519	757016	760534	1
532	*M. tuberculosis*	NZ_MMXD01000008.1	BKS17_RS03530	3519	85250	88768	1
533	*M. tuberculosis*	NZ_MKHQ01000001.1	BJF73_RS03485	3519	754918	758436	1
534	*M. tuberculosis*	NZ_MMZI01000003.1	BKR60_RS03470	3519	421511	425029	1
535	*M. tuberculosis*	NZ_MMYR01000001.1	BKR77_RS03480	3519	758954	762472	1
536	*M. tuberculosis*	NZ_MREG01000006.1	BS160_RS03605	3519	179544	183062	1
537	*M. tuberculosis*	NZ_MRFJ01000001.1	BS190_RS03590	3519	759597	763115	1
538	*M. tuberculosis*	NZ_MMWV01000006.1	BKS25_RS03520	3519	86789	90307	1
539	*M. tuberculosis*	NZ_MMYL01000001.1	BKR83_RS03480	3519	760579	764097	1
540	*M. tuberculosis*	NZ_MMKG01000001.1	BKS97_RS03500	3519	763084	766602	1
541	*M. tuberculosis*	NZ_MMJN01000001.1	BKT16_RS03460	3519	753608	757126	1
542	*M. tuberculosis*	NZ_MMTI01000002.1	BKU85_RS03495	3519	423328	426846	1
543	*M. tuberculosis*	NZ_CFVE01000001.1	AP639_RS01360	3519	276967	280485	1
544	*M. tuberculosis*	NZ_CPHY01000012.1	AQ075_RS09710	3519	56234	59752	1
545	*M. tuberculosis*	NZ_COQZ01000009.1	AQX90_RS06635	3519	73919	77437	1
546	*M. tuberculosis*	NZ_COSB01000002.1	AQX51_RS01380	3519	73939	77457	1
547	*M. tuberculosis*	NZ_CP013475.1	BTB1458_RS03470	3519	754595	758113	1
548	*M. tuberculosis*	NZ_CFUN01000002.1	AP520_RS01660	3519	76888	80406	1
549	*M. tuberculosis*	NZ_CFUH01000008.1	AP437_RS06285	3519	76893	80411	1
550	*M. tuberculosis*	NZ_COYO01000002.1	AQW21_RS01370	3519	73661	77179	1
551	*M. tuberculosis*	NZ_CPDM01000002.1	AQZ55_RS03460	3519	176019	179537	1
552	*M. tuberculosis*	NZ_FPSZ01000021.1	BT745_RS15740	3519	77092	80610	1
553	*M. tuberculosis*	NZ_FPOP01000001.1	BT427_RS01180	3519	226508	230026	1

554	*M. tuberculosis*	NZ_FQAS01000010.1	BT876_RS10665	3519	24098	27616	1
555	*M. tuberculosis*	NZ_FQAU01000019.1	BT707_RS14220	3519	74140	77658	1
556	*M. tuberculosis*	NZ_FQBA01000018.1	BT452_RS14765	3519	24107	27625	1
557	*M. tuberculosis*	NZ_FQCA01000018.1	BT525_RS15290	3519	76882	80400	1
558	*M. tuberculosis*	NZ_FPZR01000022.1	BT320_RS17585	3519	76901	80419	1
559	*M. tuberculosis*	NZ_FQOX01000006.1	BT956_RS05065	3519	17770	21288	1
560	*M. tuberculosis*	NZ_MMUD01000003.1	BKU64_RS03480	3519	419834	423352	1
561	*M. tuberculosis*	NZ_CQGA01000008.1	AEX83_RS04310	3519	70105	73623	1
562	*M. tuberculosis*	NZ_LKMF01000520.1	APD53_RS14285	3519	4521	8039	1
563	*M. tuberculosis*	NZ_CP017596.1	BJM02_03485	3519	758513	762031	1
564	*M. tuberculosis*	NZ_CFHZ01000011.1	AP776_RS06705	3519	70081	73599	1
565	*M. tuberculosis*	NZ_CNHC01000012.1	AYV76_RS06865	3519	70152	73670	1
566	*M. tuberculosis*	NZ_CNHD01000016.1	AQ095_RS08120	3519	70090	73608	1
567	*M. tuberculosis*	NZ_CHBA01000014.1	AP816_RS07260	3519	70101	73619	1
568	*M. tuberculosis*	NZ_CNCM01000012.1	AQ355_RS06410	3519	70109	73627	1
569	*M. tuberculosis*	NZ_CNKU01000015.1	AQ343_RS07615	3519	70089	73607	1
570	*M. tuberculosis*	NZ_CNBC01000046.1	AQ367_RS09480	3519	10379	13897	1
571	*M. tuberculosis*	NZ_CNBL01000011.1	AQ380_RS07350	3519	73816	77334	1
572	*M. tuberculosis*	NZ_CNBK01000015.1	AQ385_RS05135	3519	41660	45178	1
573	*M. tuberculosis*	NZ_CNCH01000014.1	AQ327_RS08025	3519	70082	73600	1
574	*M. tuberculosis*	NZ_CHAW01000007.1	AP754_RS03445	3519	70198	73716	1
575	*M. tuberculosis*	NZ_CNBD01000010.1	AQ323_RS05765	3519	70080	73598	1
576	*M. tuberculosis*	NZ_MUJT01000004.1	Mtub8_RS03080	3519	85178	88696	1
577	*M. tuberculosis*	NZ_MMCW01000001.1	BKX00_RS03475	3516	757629	761144	1
578	*M. tuberculosis*	NZ_CQRW01000007.1	AKQ63_RS04950	3519	70009	73527	1
579	*M. tuberculosis*	NZ_JUFO01000018.1	PO17_RS02980	3519	153915	157433	1
580	*M. tuberculosis*	NZ_LATP01000037.1	XM49_RS12985	3519	68943	72461	1
581	*M. tuberculosis*	NZ_LXGB01000025.1	A6455_RS10020	3519	77092	80610	1
582	*M. tuberculosis*	NZ_LGTJ01000008.1	AMQ69_RS05135	3519	68953	72471	1
583	*M. tuberculosis*	NZ_KK355765.1	W104_RS09380	3519	153904	157422	1
584	*M. tuberculosis*	NZ_KK342271.1	V958_RS17080	3519	426828	430346	1
585	*M. tuberculosis*	NZ_KK353304.1	P490_RS09155	3519	759777	763295	1
586	*M. tuberculosis*	NZ_KK353334.1	P498_RS09085	3519	763394	766912	1
587	*M. tuberculosis*	NZ_KK317029.1	AL84_RS09170	3519	759523	763041	1
588	*M. tuberculosis*	NZ_KK321276.1	AM99_RS12340	3519	85466	88984	1
589	*M. tuberculosis*	NZ_KK324243.1	AN16_RS09475	3519	153908	157426	1
590	*M. tuberculosis*	NZ_KK324544.1	AN22_RS10595	3519	179282	182800	1
591	*M. tuberculosis*	NZ_KK324845.1	AN27_RS09785	3519	85346	88864	1
592	*M. tuberculosis*	NZ_KK325001.1	AN30_RS09155	3519	758187	761705	1
593	*M. tuberculosis*	NZ_KK328195.1	K867_RS23790	3519	3157630	3161148	1
594	*M. tuberculosis*	NZ_JLPY01000011.1	T569_RS14440	3519	18177	21695	1
595	*M. tuberculosis*	NZ_AJGM01000008.1	SU1_RS0100815	3519	71487	75005	1
596	*M. tuberculosis*	NZ_AJGL01000056.1	SU3_RS0106830	3519	73367	76885	1

597	*M. tuberculosis*	NZ_AJGO01000153.1	Q7G_RS0119095	3519	39102	42620	1		
598	*M. tuberculosis*	NZ_AJGN01000088.1	SSY_RS0108665	3519	36182	39700	1		
599	*M. tuberculosis*	NZ_KK357768.1	X332_RS21355	3519	766802	770320	1		
600	*M. tuberculosis*	NZ_KK357789.1	X337_RS09095	3519	765748	769266	1		
601	*M. tuberculosis*	NZ_KK357851.1	X351_RS21375	3519	764467	767985	1		
602	*M. tuberculosis*	NZ_KK322483.1	X389_RS14945	3519	423663	427181	1		
603	*M. tuberculosis*	NZ_KK322844.1	X450_RS09090	3519	765375	768893	1		
604	*M. tuberculosis*	NZ_CCJS01000018.1	TL05_RS03545	3519	35115	38633	1		
605	*M. tuberculosis*	NZ_CNBO01000008.1	AQ238_RS04750	3519	70188	73706	1		
606	*M. tuberculosis*	NZ_CNBT01000012.1	AQ245_RS06605	3519	70161	73679	1		
607	*M. tuberculosis*	NZ_CNDR01000009.1	AQ216_RS04910	3519	69861	73379	1		
608	*M. tuberculosis*	NZ_CNFF01000011.1	AQ043_RS06840	3519	70120	73638	1		
609	*M. tuberculosis*	NZ_COSP01000011.1	AQW97_RS08230	3519	73970	77488	1		
610	*M. tuberculosis*	NZ_CPCV01000001.1	AQZ00_RS00820	3519	153657	157175	1		
611	*M. tuberculosis*	NZ_CPFE01000001.1	AQY62_RS00490	3519	87509	91027	1		
612	*M. tuberculosis*	NZ_CQGE01000005.1	AE209_RS02000	3519	11928	15446	1		
613	*M. tuberculosis*	NZ_FPSV01000008.1	BT209_RS10330	3519	125335	128853	1		
614	*M. tuberculosis*	NZ_KK320142.1	AM73_RS06830	3519	153927	157445	1		
615	*M. tuberculosis*	NZ_KK327060.1	BA36_RS12540	3519	55528	59046	1		
616	*M. tuberculosis*	NZ_KK356939.1	X178_RS21475	3519	770038	773556	1	454	5207
617	*M. tuberculosis*	NZ_LATN01000056.1	XM55_RS12775	3519	17725	21240	1		
618	*M. tuberculosis*	NZ_KK353575.1	P931_RS11060	3519	1232653	1236180	1		
619	*M. tuberculosis*	NZ_CNBX01000013.1	AQ297_RS07530	3519	70126	73644	1		
620	*M. tuberculosis*	NZ_AUTY01000456.1	N190_RS0115365	3519	4764	8282	1		
621	*M. tuberculosis*	NZ_CNBE01000011.1	AQ384_RS05105	3519	12073	15591	1		
622	*M. tuberculosis*	NZ_COSJ01000009.1	AQ397_RS07235	3519	74020	77535	1		
623	*M. tuberculosis*	NZ_CQSD01000008.1	AFA22_RS04295	3519	17734	21252	1		
624	*M. tuberculosis*	NZ_CFMF01000015.1	AP989_RS08755	3519	70116	73634	1		
625	*M. tuberculosis*	NZ_CHCR01000001.1	AP651_RS00815	3519	151726	155244	1		
626	*M. tuberculosis*	NZ_CNAT01000012.1	AQ207_RS08020"	3519	17736	21254	1		
627	*M. tuberculosis*	NZ_CNGP01000006.1	AQ048_RS03905	3519	72834	76352	1		
628	*M. tuberculosis*	NZ_KK353300.1	P489_RS09030	3513	763756	767268	1		
629	*M. tuberculosis*	NZ_MMHK01000001.1	BKO90_RS03490	3513	758642	762154	1		
630	*M. tuberculosis*	NZ_MQCN01000001.1	BOJ69_RS03590	3513	760347	763859	1		
631	*M. tuberculosis*	NZ_CNLY01000014.1	AQ311_RS08160	3519	70156	73674	1		
632	*M. tuberculosis*	NZ_CIEA01000003.1	AQ183_RS02405	3519	85260	88778	1		
633	*M. tuberculosis*	NZ_CNDS01000008.1	AYV43_RS05690	3519	70132	73650	1		
634	*M. tuberculosis*	NZ_CNKZ01000014.1	AQ148_RS07760	3519	70173	73691	1		
635	*M. tuberculosis*	NZ_MMHQ01000001.1	BKO84_RS03470	3531	757207	760737	1		
636	*M. tuberculosis*	NZ_MMVT01000001.1	BKS53_RS03490	3519	761348	764866	1		
637	*M. tuberculosis*	NZ_CQSE01000027.1	AKX79_RS10560	3517	32250	35766	1		
638	*M. tuberculosis*	NZ_CFKK01000010.1	AP729_RS05185	3519	69998	73516	1		
639	*M. tuberculosis*	NZ_CQEZ01000005.1	AFA30_RS02000	3519	63517	67035	1		

640	*M. tuberculosis*	NZ_KK353461.1	P917_RS16045	3519	153906	157424	1
641	*M. tuberculosis*	NZ_CNEC01000012.1	AQ322_RS07170	3519	70087	73605	1
642	*M. tuberculosis*	NZ_KK353493.1	P922_RS09175	3519	758446	761964	1
643	*M. tuberculosis*	NZ_MMUP01000001.1	BKS83_RS03490	3519	761558	765076	1
644	*M. tuberculosis*	NZ_CP009202.1	L791_03530	3519	759807	763325	2
645	*M. tuberculosis*	NZ_KK338904.1	N061_00698	3519	764023	767541	2
646	*M. tuberculosis*	NZ_MMVO01000001.1	BKS58_RS03480	3519	760033	763551	2
647	*M. tuberculosis*	NZ_FPXT01000014.1	BT512_RS12225	3519	76811	80329	2
648	*M. tuberculosis*	NZ_CQAO01000001.1	AEM88_RS00420	3519	74044	77562	2
649	*M. tuberculosis*	NZ_CNGD01000011.1	AQ166_RS07810	3519	72028	75546	2
650	*M. tuberculosis*	NZ_CNKF01000015.1	AQ130_RS07315	3519	70216	73734	2
651	*M. tuberculosis*	NZ_MMMB01000006.1	BKT74_RS03500	3519	421149	424667	2
652	*M. tuberculosis*	NZ_CQRU01000047.1	AFG97_RS05865	3519	4530	8048	2
653	*M. tuberculosis*	NZ_KK321058.1	AM91_01429	3519	45031	48549	2
654	*M. tuberculosis*	NZ_KK355891.1	X079_00696	3519	762467	765985	2
655	*M. tuberculosis*	NZ_KK356144.1	X104_00910	3519	149985	153503	2
656	*M. tuberculosis*	NZ_KK356505.1	X130_00699	3519	766532	770050	2
657	*M. tuberculosis*	NZ_KK356980.1	X184_00410	3519	428330	431848	2
658	*M. tuberculosis*	NZ_KK357424.1	X216_03499	3519	763645	767163	2
659	*M. tuberculosis*	NZ_KK341220.1	V460_03330	3519	3579871	3583389	2
660	*M. tuberculosis*	NZ_KK315332.1	AJ46_02475	3519	24065	27583	2
661	*M. tuberculosis*	NZ_KK313012.1	AJ16_00830	3519	17705	21223	2
662	*M. tuberculosis*	NZ_KK309686.1	AI71_RS18465	3519	17720	21238	2
663	*M. tuberculosis*	NZ_KK327500.1	BA57_RS18980	3519	88934	92452	2
664	*M. tuberculosis*	NZ_KK339365.1	P424_RS09025	3519	764815	768333	2
665	*M. tuberculosis*	NZ_MRFP01000002.1	BS196_RS03580	3519	419107	422625	2
666	*M. tuberculosis*	NZ_MMHF01000001.1	BKO95_RS03505	3519	761542	765060	2
667	*M. tuberculosis*	NZ_MMGB01000002.1	BKP25_RS03490	3519	423035	426553	2
668	*M. tuberculosis*	NZ_MMYC01000001.1	BKR92_RS03490	3519	761767	765285	2
669	*M. tuberculosis*	NZ_MMSC01000007.1	BKV17_RS03515	3519	87612	91130	2
670	*M. tuberculosis*	NZ_MMGJ01000001.1	BKP17_RS03480	3519	757526	761044	2
671	*M. tuberculosis*	NZ_MMQF01000008.1	BKV67_RS03520	3519	135987	139505	2
672	*M. tuberculosis*	NZ_MMXC01000012.1	BKS18_RS03535	3519	85166	88684	2
673	*M. tuberculosis*	NZ_COTL01000010.1	AQX27_RS08450	3519	85264	88782	2
674	*M. tuberculosis*	NZ_CPXC01000002.1	AFE92_RS02050	3519	152460	155978	2
675	*M. tuberculosis*	NZ_CGEM01000001.1	AP563_RS00420	3519	74025	77543	2
676	*M. tuberculosis*	NZ_CGCA01000019.1	AQG61_RS08480	3519	42927	46445	2
677	*M. tuberculosis*	NZ_MQFV01000001.1	BOK83_RS03595	3519	761561	765079	2
678	*M. tuberculosis*	NZ_MQCI01000001.1	BOJ64_RS03595	3519	761499	765017	2
679	*M. tuberculosis*	NZ_KK354745.1	W035_00698	3519	761825	765343	2
680	*M. tuberculosis*	NZ_KK355361.1	W079_01369	3519	89446	92964	2
681	*M. tuberculosis*	NZ_KK354240.1	V989_RS09120	3519	781081	784599	2
682	*M. tuberculosis*	NZ_KK353341.1	P499_RS21720	3519	3576229	3579747	2

683	*M. tuberculosis*	NZ_KK328036.1	K848_RS12970	3519	764409	767927	2
684	*M. tuberculosis*	NZ_KK353294.1	P488_RS09135	3522	760422	763943	2
685	*M. tuberculosis*	NZ_MMOR01000002.1	BKW07_RS03500	3522	423087	426608	2
686	*M. tuberculosis*	NZ_KK356137.1	X103_RS09280	3519	772074	775592	2
687	*M. tuberculosis*	NZ_KK356525.1	X133_RS09185	3519	758677	762195	2
688	*M. tuberculosis*	NZ_KK356917.1	X171_RS09220	3519	767400	770918	2
689	*M. tuberculosis*	NZ_KK357325.1	X211_RS09200	3519	760268	763786	2
690	*M. tuberculosis*	NZ_CP002885.1	CFBR_RS03490	3519	758630	762148	2
691	*M. tuberculosis*	NZ_KK337379.1	AP46_RS08085	3519	179430	182948	2
692	*M. tuberculosis*	NZ_KK353738.1	P956_RS07485	3519	358793	362311	2
693	*M. tuberculosis*	NZ_KK353773.1	P960_RS12040	3519	1389462	1392980	2
694	*M. tuberculosis*	NZ_KK322146.1	AN64_RS09220	3519	763665	767183	2
695	*M. tuberculosis*	NZ_KK322269.1	AN70_RS09165	3519	763345	766863	2
696	*M. tuberculosis*	NZ_KK312387.1	AJ04_RS07710	3519	152714	156232	2
697	*M. tuberculosis*	NZ_KK339400.1	Q624_02964	3519	3190600	3194118	2
698	*M. tuberculosis*	NZ_CFMP01000001.1	AP502_RS02055	3519	449431	452949	2
699	*M. tuberculosis*	NZ_MMXS01000001.1	BKS02_RS03460	3519	753859	757377	2
700	*M. tuberculosis*	NZ_MRFG01000001.1	BS187_RS03595	3519	761627	765145	2
701	*M. tuberculosis*	NZ_MRFI01000002.1	BS189_RS03600	3519	426165	429683	2
702	*M. tuberculosis*	NZ_COHO01000002.1	AYU03_RS01780	3519	152469	155987	2
703	*M. tuberculosis*	NZ_CONB01000002.1	AQV24_RS01930	3519	125432	128950	2
704	*M. tuberculosis*	NZ_CPYX01000003.1	AEM17_RS03420	3519	152524	156042	2
705	*M. tuberculosis*	NZ_FPRR01000017.1	BT310_RS12020	3519	17773	21291	2
706	*M. tuberculosis*	NZ_FQOT01000018.1	BT975_RS13925	3519	74121	77639	2
707	*M. tuberculosis*	NZ_FQBT01000011.1	BT687_RS10200	3519	17770	21288	2
708	*M. tuberculosis*	NZ_CIIX01000013.1	AP860_RS07650	3519	72877	76395	2
709	*M. tuberculosis*	NZ_MQEU01000001.1	BOK28_RS03580	3519	758648	762166	2
710	*M. tuberculosis*	NZ_CP010968.1	UZ39_RS05595	3519	1222808	1226326	2
711	*M. tuberculosis*	NZ_KK355732.1	W098_RS07715	3519	424902	428420	2
712	*M. tuberculosis*	NZ_KK353290.1	P487_RS19365	3519	758318	761836	2
713	*M. tuberculosis*	NZ_JJXY01000001.1	AL88_RS23280	3519	3938428	3941946	2
714	*M. tuberculosis*	NZ_KK320966.1	AM89_RS08710	3519	152763	156281	2
715	*M. tuberculosis*	NZ_KK322478.1	X388_RS22715	3519	125650	129168	2
716	*M. tuberculosis*	NZ_CNHV01000010.1	AQ030_RS06085	3519	73100	76618	2
717	*M. tuberculosis*	NZ_CNBP01000012.1	AQ206_RS06980	3519	70112	73630	2
718	*M. tuberculosis*	NZ_CNCB01000014.1	AQ293_RS07475	3519	71276	74794	2
719	*M. tuberculosis*	NZ_COUS01000002.1	AQV10_RS01965	3519	136652	140170	2
720	*M. tuberculosis*	NZ_KK328297.1	K879_RS13085	3519	762700	766218	2
721	*M. tuberculosis*	NZ_KK328754.1	AO04_RS09195	3519	762737	766255	2
722	*M. tuberculosis*	NZ_KK355946.1	X090_RS22890	3519	764659	768177	2
723	*M. tuberculosis*	NZ_KK357335.1	X212_RS09210	3519	768833	772351	2
724	*M. tuberculosis*	NZ_MRFQ01000001.1	BS197_RS03580	3519	757611	761129	2
725	*M. tuberculosis*	NZ_KK357944.1	X379_RS12225	3519	329269	332787	2

726	*M. tuberculosis*	NZ_MQGL01000003.1	BOK99_RS03600	3519	421731	425249	2
727	*M. tuberculosis*	NZ_COXB01000009.1	AQ405_RS07730	3507	73991	77497	2
728	*M. tuberculosis*	NZ_MCQX01000019.1	BHQ12_RS03535	3519	84492	88010	2
729	*M. tuberculosis*	NZ_KK341495.1	V739_00622	3519	284590	288108	3
730	*M. tuberculosis*	NZ_CNCX01000010.1	AQ340_RS05440	3519	70196	73714	3
731	*M. tuberculosis*	NZ_MMCT01000004.1	BKX03_RS03505	3519	423658	427176	3
732	*M. tuberculosis*	NZ_KK307638.1	Z568_02292	3519	24097	27615	3
733	*M. tuberculosis*	NZ_KK356255.1	X114_00352	3519	152635	156153	3
734	*M. tuberculosis*	NZ_KK353475.1	P919_RS09170	3519	757375	760893	3
735	*M. tuberculosis*	NZ_KK339360.1	N133_01734	3519	1868156	1871674	3
736	*M. tuberculosis*	NZ_MMKH01000005.1	BKS96_RS03505	3519	422998	426516	3
737	*M. tuberculosis*	NZ_MMJZ01000002.1	BKT04_RS03495	3519	423025	426543	3
738	*M. tuberculosis*	NZ_MMSP01000017.1	BKV04_RS03535	3519	84671	88189	3
739	*M. tuberculosis*	NZ_MMQT01000004.1	BKV52_RS03490	3519	421751	425269	3
740	*M. tuberculosis*	NZ_FPMY01000018.1	BT552_RS14715	3519	73143	76661	3
741	*M. tuberculosis*	NZ_FPTG01000007.1	BT514_RS07315	3519	125577	129095	3
742	*M. tuberculosis*	NZ_MQKB01000004.1	BOM07_RS03625	3519	424424	427942	3
743	*M. tuberculosis*	NZ_KL405716.1	X126_RS09260	3519	764603	768121	3
744	*M. tuberculosis*	NZ_KK356651.1	X156_RS07600	3519	426471	429989	3
745	*M. tuberculosis*	NZ_KK357440.1	X220_RS09090	3519	758548	762066	3
746	*M. tuberculosis*	NZ_KK353409.1	P912_RS09215	3519	764213	767731	3
747	*M. tuberculosis*	NZ_KK311356.1	AI93_RS18095	3519	17769	21287	3
748	*M. tuberculosis*	NZ_KK325272.1	BA06_RS16725	3519	137784	141302	3
749	*M. tuberculosis*	NZ_MMQG01000013.1	BKV66_RS03520	3519	85212	88730	3
750	*M. tuberculosis*	NZ_FPRA01000014.1	BT592_RS13295	3519	24106	27624	3
751	*M. tuberculosis*	NZ_CNDW01000013.1	AQ301_RS06660	3519	70053	73571	3
752	*M. tuberculosis*	NZ_CFNF01000001.1	AP624_RS01095	3519	217453	220971	3
753	*M. tuberculosis*	NZ_FPPD01000010.1	BT562_RS11475	3519	125407	128925	3
754	*M. tuberculosis*	NZ_KK318322.1	AM35_RS11305	3519	152662	156177	3
755	*M. tuberculosis*	NZ_KK322898.1	Z522_RS06925	3519	152736	156254	3
756	*M. tuberculosis*	NZ_KK340754.1	V202_RS08245	3519	152739	156257	3
757	*M. tuberculosis*	NZ_MMRG01000003.1	BKV39_RS03500	3519	422962	426480	3
758	*M. tuberculosis*	NZ_KK339126.1	N096_00698	3519	766022	769540	4
759	*M. tuberculosis*	NZ_MMKR01000002.1	BKS86_RS03490	3519	422881	426399	4
760	*M. tuberculosis*	NZ_CRZH01000015.1	AKS79_RS05575	3519	12127	15645	4
761	*M. tuberculosis*	NZ_KK316690.1	AL32_00652	3519	153914	157432	4
762	*M. tuberculosis*	NZ_KK356482.1	X124_00694	3519	756340	759858	4
763	*M. tuberculosis*	NZ_KK339346.1	N131_RS07670	3519	427353	430871	4
764	*M. tuberculosis*	NZ_KK315141.1	AJ44_00556	3519	153909	157427	4
765	*M. tuberculosis*	NZ_KK312008.1	AJ00_02218	3519	17737	21255	4
766	*M. tuberculosis*	NZ_CPGO01000004.1	AQZ04_RS03970	3519	152366	155884	4
767	*M. tuberculosis*	NZ_MMWC01000001.1	BKS44_RS03470	3519	755442	758960	4
768	*M. tuberculosis*	NZ_KK351749.1	P463_RS11205	3519	152087	155605	4

769	*M. tuberculosis*	NZ_JOKR01000009.1	HA35_RS26360	3519	73693	77211	4
770	*M. tuberculosis*	NZ_MMJA01000003.1	BKT29_RS03485	3519	179544	183062	4
771	*M. tuberculosis*	NZ_CNDP01000011.1	AQ316_RS07830	3519	70191	73709	4
772	*M. tuberculosis*	NZ_KK341763.1	V915_RS20775	3519	759296	762814	4
773	*M. tuberculosis*	NZ_KK356575.1	X142_00696	3519	761414	764932	5
774	*M. tuberculosis*	NZ_KK351010.1	P462_03578	3519	3867166	3870684	5
775	*M. tuberculosis*	NZ_KK308427.1	Z579_00852	3519	153854	157372	5
776	*M. tuberculosis*	NZ_MMZS01000001.1	BKR50_RS03490	3519	761486	765004	5
777	*M. tuberculosis*	NZ_MMVV01000001.1	BKS51_RS03495	3519	760974	764492	5
778	*M. tuberculosis*	NZ_FPXS01000003.1	BT529_RS04795	3519	154441	157959	5
779	*M. tuberculosis*	NZ_FQBC01000001.1	BT500_RS00720	3519	126664	130182	5
780	*M. tuberculosis*	NZ_COJJ01000002.1	AQT65_RS01385	3519	73974	77492	5
781	*M. tuberculosis*	NZ_KK317077.1	AM01_RS09205	3519	774875	778393	5
782	*M. tuberculosis*	NZ_KK329857.1	AO39_RS08095	3519	198235	201753	5
783	*M. tuberculosis*	NZ_CM002022.1	M208_RS03575	3519	759675	763193	6
784	*M. tuberculosis*	NZ_MMZX01000001.1	BKR45_RS03485	3519	760668	764186	6
785	*M. tuberculosis*	NZ_MMXJ01000002.1	BKS11_RS03495	3519	422909	426427	6
786	*M. tuberculosis*	NZ_MMWK01000008.1	BKS36_RS03515	3519	136042	139560	6
787	*M. tuberculosis*	NZ_MMLP01000003.1	BKT86_RS03485	3519	85253	88771	6
788	*M. tuberculosis*	NZ_MRER01000001.1	BS172_RS03585	3519	758730	762248	6
789	*M. tuberculosis*	NZ_MMRX01000009.1	BKV22_RS03520	3519	136013	139531	6
790	*M. tuberculosis*	NZ_FPNS01000010.1	BT215_RS09310	3519	24106	27624	6
791	*M. tuberculosis*	NZ_KK317085.1	AM04_RS11115	3519	1225444	1228962	6
792	*M. tuberculosis*	NZ_KK353635.1	P940_00408	3519	424679	428197	7
793	*M. tuberculosis*	NZ_MMZO01000002.1	BKR54_RS03515	3519	425673	429191	7
794	*M. tuberculosis*	NZ_FPXK01000009.1	BT848_RS07535	3519	24106	27624	7
795	*M. tuberculosis*	NZ_CFTK01000001.1	AP418_RS00680	3519	125411	128929	7
796	*M. tuberculosis*	NZ_KK356245.1	X110_00696	3519	763807	767325	8
797	*M. tuberculosis*	NZ_KK321917.1	AN59_RS18950	3519	763120	766638	8
798	*M. tuberculosis*	NZ_CNDT01000007.1	AQ317_RS03650	3519	69922	73440	9
799	*M. tuberculosis*	NZ_KK338039.1	AP58_00163	3519	153952	157470	9
800	*M. tuberculosis*	NZ_MMPA01000001.1	BKV98_RS03475	3519	757604	761122	9
801	*M. tuberculosis*	NZ_KK357573.1	X223_RS17085	3519	765910	769428	9
802	*M. tuberculosis*	NZ_KK353539.1	P926_RS07665	3519	426515	430033	10
803	*M. tuberculosis*	NZ_MMGI01000001.1	BKP18_RS03485	3519	758954	762472	10
804	*M. tuberculosis*	NZ_FPRK01000007.1	BT369_RS06085	3519	125623	129141	10
805	*M. tuberculosis*	NZ_CM002054.1	M391_RS03610	3519	761210	764728	10
806	*M. tuberculosis*	NZ_FPOX01000003.1	BT714_RS03625	3519	125215	128733	11
807	*M. tuberculosis*	NZ_LJIK01000204.1	AN949_RS12090	3519	12099	15617	11
808	*M. tuberculosis*	NZ_KK354536.1	W018_01124	3519	1226014	1229532	11
809	*M. tuberculosis*	NZ_KK327698.1	X161_00405	3519	425992	429510	12
810	*M. tuberculosis*	NZ_KK338911.1	N063_00699	3519	764381	767899	12
811	*M. tuberculosis*	NZ_CM000788.2	CMC_RS03645	3519	760585	764103	13

812	*M. tuberculosis*	NZ_KK327723.1	X164_00698	3519	766809	770327	13		
813	*M. tuberculosis*	NZ_MMWX01000004.1	BKS23_RS03510	3519	136079	139597	13		
814	*M. tuberculosis*	NZ_FQOJ01000019.1	BT963_RS15220	3519	24103	27621	13		
815	*M. tuberculosis*	NZ_KK356570.1	X139_03227	3519	761550	765068	14		
816	*M. tuberculosis*	NZ_MMSM01000001.1	BKV07_RS03495	3519	761561	765079	14		
817	*M. tuberculosis*	NZ_KK311070.1	AI90_00652	3519	153909	157427	17		
818	*M. tuberculosis*	NZ_MMHR01000001.1	BKO83_RS03475	3519	756659	760177	18		
819	*M. tuberculosis*	NZ_KK355971.1	X094_00697	3519	768491	772009	18		
820	*M. tuberculosis*	NZ_KK339271.1	N117_RS09080	3519	763682	767200	19		
821	*M. tuberculosis*	NZ_KK356908.1	X169_00697	3519	758946	762464	19		
822	*M. tuberculosis*	NZ_KK354446.1	W010_RS08990	3519	763504	767022	21		
823	*M. tuberculosis*	NZ_KK356487.1	X127_RS09260	3519	765905	769423	21		
824	*M. tuberculosis*	NZ_MMQJ01000007.1	BKV62_RS03515	3519	86848	90366	22		
825	*M. tuberculosis*	NZ_KK307203.1	Z559_00697	3519	762187	765705	23		
826	*M. tuberculosis*	NZ_KK356621.1	X151_00406	3519	425922	429440	23		
827	*M. tuberculosis*	NZ_MMYJ01000001.1	BKR85_RS03485	3519	758973	762491	24		
828	*M. tuberculosis*	NZ_KK341412.1	V738_RS08605	3519	136413	139931	31		
829	*M. tuberculosis*	NZ_KK357076.1	X189_RS07625	3519	426970	430488	55		
830	*M. tuberculosis*	NZ_MMHS01000001.1	BKO82_RS03470	3519	757122	760640	68		
831	*M. tuberculosis*	NZ_CM000789.2	CME_RS03635	3519	760585	764103	69		
832	*M. tuberculosis*	NC_021740.1	M943_03465	3519	759818	763336	82		
833	*M. tuberculosis*	NZ_KK356528.1	X134_00328	3519	327755	331273	87		
834	*M. tuberculosis*	NZ_KK308613.1	Z581_02193	3519	24087	27605	92		
835	*M. tuberculosis*	NZ_CM002053.1	M390_RS03645	3519	762498	766016	93		
836	*M. tuberculosis*	NZ_KK355966.1	X093_00699	3519	766991	770509	125		
837	*M. tuberculosis*	NZ_KK356612.1	X149_RS09165	3519	758928	762446	201		
838	*M. tuberculosis*	NZ_KK307972.1	Z574_00163	3519	153295	156813	217		
839	*M. tuberculosis*	NC_020089.1	MT7199_RS03510	3519	764552	768070	264		
840	*M. tuberculosis*	NZ_KK341230.1	V736_00362	3519	152742	156260	456		
841	*M. tuberculosis*	NZ_KK354898.1	W052_RS22065	3519	3667852	3671370	579		
842	*M. tuberculosis*	NZ_JLCZ01000003.1	BM97_RS09330	3519	152739	156257	606		
843	*M. tuberculosis*	NC_000962.3	Rv0667	3519	759807	763325	1025		
844	*M. tusciae*	NZ_KI912270.1	MYCTUDRAFT_RS0216720	3504	2994788	2998291	1	2	2
845	*M. tusciae*	NZ_MVIM01000003.1	BST47_RS06570	3501	138724	142224	1		
846	*M. ulcerans*	NZ_MDUB01000469.1	A3649_RS23095	3531	73586	77116	1	1	1
847	*M. vaccae*	NZ_CP011491.1	MYVA_RS05200	3513	1109894	1113406	3	1	3
848	*M. vanbaalenii*	NC_008726.1	MVAN_RS06355	3504	1344369	1347872	1	1	1
849	*M. vulneris*	NZ_MBDY01000027.1	A5717_RS08565	3510	33255	36764	1		
850	*M. vulneris*	NZ_MBEF01000022.1	A5721_RS06770	3510	132612	136121	1		
851	*M. vulneris*	NZ_NCXM01000019.1	B8W69_RS18610	3462	83763	87224	1	5	6
852	*M. vulneris*	NZ_CCBG010000001.1	BN979_RS04990	3510	1024587	1028096	1		
853	*M. vulneris*	NZ_MBEH01000061.1	A5729_RS18160	3510	133263	136772	2		
854	*M. wolinskyi*	NZ_LQQA01000030.1	AWC31_RS24275	3510	138247	141756	1	2	2

855	*M. wolinskyi*	NZ_LGTW01000024.1	AFM11_RS28830	3510	122760	126269	1	2	2
856	*M. xenopi*	NZ_AJFI01000116.1	MXEN_RS21130	3507	1502	5008	1	2	2
857	*M. xenopi*	NZ_LQQB01000145.1	AWC32_RS22210	3525	61130	64654	1		
858	*M. yongonense*	NZ_MBDX01000208.1	A5644_RS18490	3462	5436	8897	1		
859	*M. yongonense*	NZ_MBDZ01000168.1	A5689_RS13755	3462	16888	20349	1	3	4
860	*M. yongonense*	NZ_CP015965.1	MOTT27_RS21565	3522	4576649	4580170	2		
